# Unique Glutelin Expression Patterns and Seed Endosperm Structure Facilitate Glutelin Accumulation in Polyploid Rice Seed

**DOI:** 10.1186/s12284-021-00500-0

**Published:** 2021-07-05

**Authors:** Lu Gan, Baosheng Huang, Zhaojian Song, Yachun Zhang, Yujie Zhang, Si Chen, Liqi Tong, Zhisong Wei, Lingxiang Yu, Xiangbo Luo, Xianhua Zhang, Detian Cai, Yuchi He

**Affiliations:** 1 State Key Laboratory of Biocatalysis and Enzyme Engineering, School of Life Sciences, Hubei University, Wuhan, China; 2 School of Chemistry & Environmental Engineering, Hanjiang Normal University, Shiyan, China; 3 Wuhan Polyploid Biology Technology Co. Ltd, Wuhan, China

**Keywords:** Seed storage protein, Glutelin, Polyploidization, Expression analysis, Cytohistological analysis

## Abstract

**Background:**

Rice is not only an essential food but also a source of high quality protein. Polyploidy is an evolutionary trajectory in plants, and enhancing glutelin by polyploidization is an attractive strategy for improving the nutritional value of rice seeds and presents a great potential for enhancing the commercial value of rice. Elucidating the mechanisms underlying glutelin synthesis and accumulation in tetraploid rice is of great significance.

**Results:**

To enhance the nutritional value of rice, we developed tetraploid rice and evaluated the contents of various nutrient elements in mature seeds. The results revealed a significant increase in protein contents, including the total seed storage proteins, glutelins, and amino acids in tetraploid rice when compared with those in diploid rice. Tandem mass tag-based quantitative proteomic analyses of seeds revealed that glutelins regulated by several glutelin genes in 9311-4x were significantly up-regulated (≥1.5-fold), which was further verified by immunoblot analyses. In addition, temporal expression patterns of various glutelin subunits in different rice lines were investigated. The results revealed significant differences in the expression patterns between diploid and tetraploid rice seeds. Cytohistological analyses results revealed that the thickness of aleurone cell layers increased significantly by 32% in tetraploid rice, the structures of protein storage vacuoles (PSVs) in sub-aleurone cells were more diverse and abundant than those of diploid rice. Temporal expression and proteomic analyses results revealed that protein disulfide isomerase-like 1–1 expression levels were higher in tetraploid rice than in diploid rice, and that the gene responded to oxidative folding with increased levels of proglutelin and appropriate distribution of seed glutelins in tetraploid rice.

**Conclusion:**

The results of the present study revealed that polyploidization increased glutelin content by influencing glutelin biosynthesis, transport, and deposition, while variations in glutelin accumulation between tetraploid and diploid rice were largely manifested in the initial time, duration, and relative levels of various glutelin gene expressions during seed filling stages. These findings provide novel insights into improving the protein quality and nutritional value of rice seeds by polyploid breeding.

**Supplementary Information:**

The online version contains supplementary material available at 10.1186/s12284-021-00500-0.

## Background

Seed storage proteins (SSPs) are the second most abundant components of rice seeds, next to starch, accounting for approximately 7%–10% of the seed weight, and are key factors influencing the nutritional quality, pasting, and textural properties of cooked rice (Kawakatsu et al. 2010). SSPs provide a nitrogen source for seed germination, seedling development and a nutrient source for humans (Juliano 1993; Kawakatsu et al. 2010). Especially, rice glutelin is a high-quality plant protein with a higher nutritional value than other cereal proteins (Friedman 1996). Therefore, we tried to improve rice protein content and nutritional quality by enhancing glutelin and amino acid contents. SSPs are classified into glutelins, prolamins, globulins, and albumins based on their solubility characteristics in the extraction solvents (Kawakatsu et al. 2008). Rice seeds accumulate glutelins as key SSPs, and they account for 60–80%, followed by prolamins (5%–10%) (Shewry and Halford 2002). Prolamin is indigestible and reduces the nutritional quality of rice protein due to the hydrophobic nature of its structure (Kubota et al. 2010). Glutelins in rice seeds are high-quality plant proteins containing several essential amino acids that can be easily digested and absorbed compared to prolamins (Friedman 1996). Therefore, breeding of high-quality rice can be achieved by enhancing rice glutelins. Several studies have been conducted on the mechanisms of glutelin biosynthesis and accumulation in diploid rice (Kawakatsu et al. 2010; Kim et al. 2012b; Lee et al. 2015). However, few studies have focused on the potential variations in molecular and cellular traits in tetraploid rice. A series of polyploid meiosis stability tetraploid rice lines, which exhibit stable meiotic features and high seed set rates, such as A3-4x and CX35-4x have been developed (Cai et al. 2007; Song et al. 2007; Tu et al. 2014; Xiong et al. 2019; Koide et al. 2020). Furthermore, the findings of previous studies suggested that polyploidy could enhance yield and environmental adaptability of rice (Song et al. 2007; Tu et al. 2014). Notably, significant increases in glutelins and total proteins were observed in 24 tetraploid rice lines when compared with diploid lines, while other related properties such as amylose contents did not exhibit similar increases (Table S5). Therefore, elucidating the mechanisms underlying glutelin synthesis and accumulation in tetraploid rice is of great significance.

Glutelins in diploid rice are represented by a multigene family in rice plants and to date, 18 full-length genes annotated as rice glutelins have been investigated (*Oryza sativa* L. cv. Nipponbare; Table S1), and multiple sequence alignments between amino acid sequences of rice glutelins have been performed using MEGA X (Fig. S1). Glutelin proteins are classified into three groups and four sub-families based on the amino acid sequence similarities: *GluA, GluB, GluC*, *and GluD* subfamilies. Previous studies have revealed that glutelins and globulins are deposited into irregularly-shaped protein bodies II (PB-IIs) derived from protein storage vacuoles (PSVs) (Krishnan et al. 1992; Kumamaru et al. 2010), whereas prolamins are stored in spherical protein bodies I (PB-Is), derived from the endoplasmic reticulum (ER) (Tanaka et al. 1980; Krishnan et al. 1986; Saito et al. 2012). Glutelins comprise of three subunits including 57-kDa glutelin precursors, 37-kDa acidic, and 20-kDa basic glutelin subunits (Tanaka et al. 1980; Yamagata and Tanaka 1986). The 57-kDa glutelin precursor is initially synthesized in the ER, and subsequently transported to the PSVs through the Golgi apparatus and the dense vesicle-mediated post-Golgi trafficking pathway ultimately form mature 37-kDa acidic and 20-kDa basic glutelin subunits by special cleavage of vacuolar processing enzymes in rice endosperms (Tanaka et al. 1980; Kumamaru et al. 2010; Ren et al. 2014). Defects during the transfer process of proglutelins before they reach the PB-II can lead to overaccumulation of the 57-kDa glutelin precursor proteins and insufficient synthesis of glutelin subunits in the seeds (Wang et al. 2016). Shear ripening of glutelins is essential for protein crystallization and maintenance of PB-II morphology (Kumamaru et al. 2010). The increase in glutelins is primarily manifested by the increase in glutelin subunits after polyploidization. However, the mechanism via which the storage proteins are initially exported from the ER remains unknown. Therefore, it is imperative to explore the dynamic expression patterns and the mechanisms of regulation of glutelins in tetraploid rice.

Tetraploid rice exhibit distinct variations in panicle length, seed size and 1000-seed weight (Song et al. 2007). The endosperm accounts for 80%–90% of the rice seed. An outer aleurone layer and an inner starchy endosperm constitute the bulk of the cereal endosperm. The aleurone layers predominantly accumulate storage proteins, lipids, vitamins, and minerals (Becraft and Yi 2011; Wu et al. 2016). Most cereal seeds have single-cell-layered aleurones, except rice (*Oryza sativa*) and barley (*Hordeum vulgare*) (Jestin et al. 2008; Becraft and Yi 2011). Rice aleurone layer structure is variable, it is predominantly a single cell layer but could consist of three or four cell layers in a small, thickened region adjacent to the dorsal vascular bundle (Jestin et al. 2008; Wu et al. 2016). A unique structural sub-aleurone layer is located in the outermost layer of a starch-filled endosperm in which a few starch granules and numerous protein bodies (PBs) can be observed. Mutations of a DEK1 homolog, ADL1, or suppressed expression of OsCR4 in rice cause variations in the degree of aleurone cell layer loss in rice endosperms (Shen et al. 2003; Kawakatsu et al. 2009). Mutations of DNA demethylase OsROS1 or NAKED ENDOSPERM transcription factors lead to multiple aleurone cell layers, and significant increases in proteins, lipids, vitamins, minerals, and dietary fibers have been observed with an increase in the thickness of aleurone cell layers (Yi et al. 2015; Liu et al. 2018). In the present study, the findings have demonstrated that polyploidization alters the thickness of the aleurone cell layer and sub-aleurone layer structure, including protein bodies and starch granules, which could be associated with the variations in protein content. Therefore, it is critical to investigate variations in the structure of the aleurone layer and starchy endosperm between diploid and tetraploid rice, and to determine the potential cytological factors facilitating the increase in protein contents.

Enhancing glutelin by polyploidization is an attractive target for improving the nutritional value of rice seeds. Various detection methods have been used to explore variations in SSPs contents in tetraploid rice, especially glutelin. Glutelin composition supported the hypothesis that polyploidization can enhance the nutritional value of rice. In the present study, individual glutelin subunits of indica rice cv. 9311-2x and 9311-4x were isolated using sodium dodecyl sulfate-polyacrylamide gel electrophoresis (SDS-PAGE), and identified using tandem mass tags (TMT) to enhance our understanding of the mechanisms of rice glutelin accumulation in tetraploid rice. Based on the subunit identification results, variations between dynamic accumulations of glutelins were studied by immunoblotting and gene expression analyses. In addition, cytohistological analyses of aleurone structures were performed under light microscopy (LM), scanning electron microscopy (SEM), and transmission electron microscopy (TEM). The results revealed that polyploidization increased glutelin content by influencing glutelin biosynthesis, transport, and deposition, while variations in glutelin accumulation between tetraploid and diploid rice were largely manifested in the initial time, duration, and relative levels of various glutelin gene expressions during seed filling stages. Glutelin biosynthesis in tetraploid rice was delayed by 2 days and up-regulated during specific periods, with results indicating that active accumulation duration of glutelin was 6 days longer than that of diploid rice. Meanwhile, the observation of deformed PSVs suggested that polyploidization induced morphological changes and considerable quantitative variations in the PSVs, which led to the increase in glutelin contents in rice seeds. Overall, the results could provide novel insights into the biosynthesis and accumulation patterns of glutelin in tetraploid rice, which explains the increase in protein content in tetraploid rice, and could be a potential breeding approach for improving the nutritional value of rice seeds.

## Results

### Polyploidization Alters the Protein Content of Mature Seeds

**Fig. 1:**
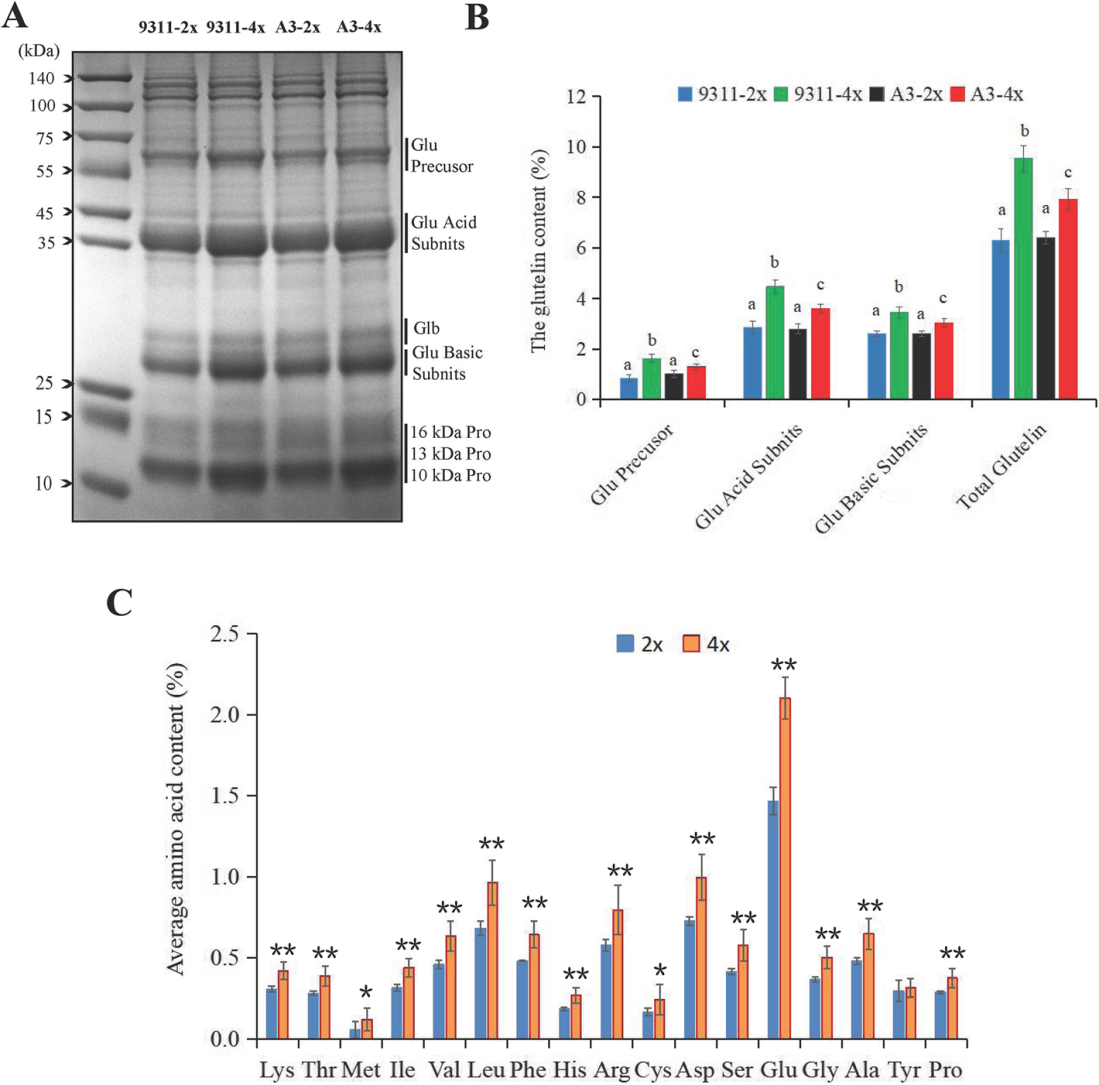
Expression analyses of SSPs and amino acids in mature seeds of brown rice (9311 and A3, harvested in 2019). **A** Total proteins in brown rice (9311 and A3) were separated by SDS-PAGE (4%–20% gradient gel). The vertical lines represent protein bands with varying intensities between tetraploid and diploid rice seeds. M = molecular size marker. **B** SDS-PAGE gel bands were scanned and analyzed using Image J software. Different lowercase letters indicate significant differences (*p* < 0.05) as determined by one-way ANOVA and Tukey’s test. **C** Average content of amino acid compositions in mature seeds of tetraploid and diploid rice (9311 and A3). Significant differences in amino acid contents between tetraploid and diploid rice seeds were tested using independent Student’s *t*-test (∗*p* < 0.05, ∗∗*p* < 0.01). Values are presented as means ± standard deviation (SD, error bars) of three replicates

The contents of various nutrient elements in mature seeds from 24 pairs of tetraploid and diploid rice were evaluated to determine whether whole-genome duplication (WGD) influenced the nutritional value of rice. The results revealed that total brown rice protein, glutelin, and amino acid contents in tetraploid rice seeds increased considerably compared with diploid rice seeds in 2018 and 2019 (Fig. 1, Tables S2 and S3), although the extent of alteration was genotype-dependent. Among them, seven pairs of tetraploid and diploid rice were selected and separated by SDS-PAGE to further verify variation in protein contents (Fig. 1, Fig. S2).

We extracted total storage proteins from powdered mature brown rice seeds with seven pairs of rice genotypes in 2019, which were subsequently analyzed using SDS-PAGE (4%–20% gradient gel) to verify if the increase in total protein content was primarily attributed to increases in the synthesis and accumulation of glutelins (Fig. 1A and B, Fig. S2). Glutelins and prolamins were extracted and evaluated independently (Table S3). Glutelin contents in all tetraploid rice increased at varying degrees when compared with the contents in diploid rice (Fig. 1A and B, Table S3). SDS-PAGE gel bands of two pairs of cultivated varieties (9311-2x and 9311-4x, A3-2x and A3-4x) were mainly analyzed using Image J software. The quantities of 57-kDa proglutelin (up 94.69%), and 37-kDa acidic (56.67%) and 20-kDa basic subunits (32.49%) in tetraploid rice seeds (9311-4x) were significantly higher than the quantities in diploid rice seeds (9311-2x; Fig. 1A and B). Similar expression levels were observed in A3-2x and A3-4x. The results revealed that polyploidization largely influenced the total protein content by altering glutelin synthesis. Furthermore, WGD exerted a greater effect on storage protein contents in 9311 than that in the other cultivars (Fig. 1A and B, Table S3).

Most recent studies have focused on enhancing protein utilization efficiency by increasing glutelin contents (Yoon et al. 2012). Glutelin contains substantial amounts of lysine and other essential amino acids. Lysine, which is a primary limiting amino acid, influences the nutritional quality of rice consumed by animals and human beings. The amino acid contents of mature seeds in two pairs of rice varieties (9311-2x and 9311-4x, A3-2x and A3-4x) were determined to investigate the effect of polyploidization on amino acids associated with total SSPs. A total of 17 amino acid types in tetraploid rice were substantially up-regulated when compared with the amino acid contents in diploid rice, excluding tyrosine from A3 (Fig. 1C, Table S4). The total amino acid and total protein contents exhibited a similar trend, increasing by 58% on average (Tables S2 and S4), which demonstrated the reliability of the experimental results. In addition, analyses of 9311-2x and 9311-4x revealed that various amino acid contents in tetraploid rice increased by 49%–75% when compared with those in diploid rice, whereas the levels of the primary limiting amino acid (lysine) increased by 49.9% (Table S4). The results suggested that tetraploid rice was more nutritious than diploid rice to a certain extent.

### Differentially Expressed Glutelin Profiles in Mature Seeds Between Tetraploid and Diploid Rice

**Fig. 2:**
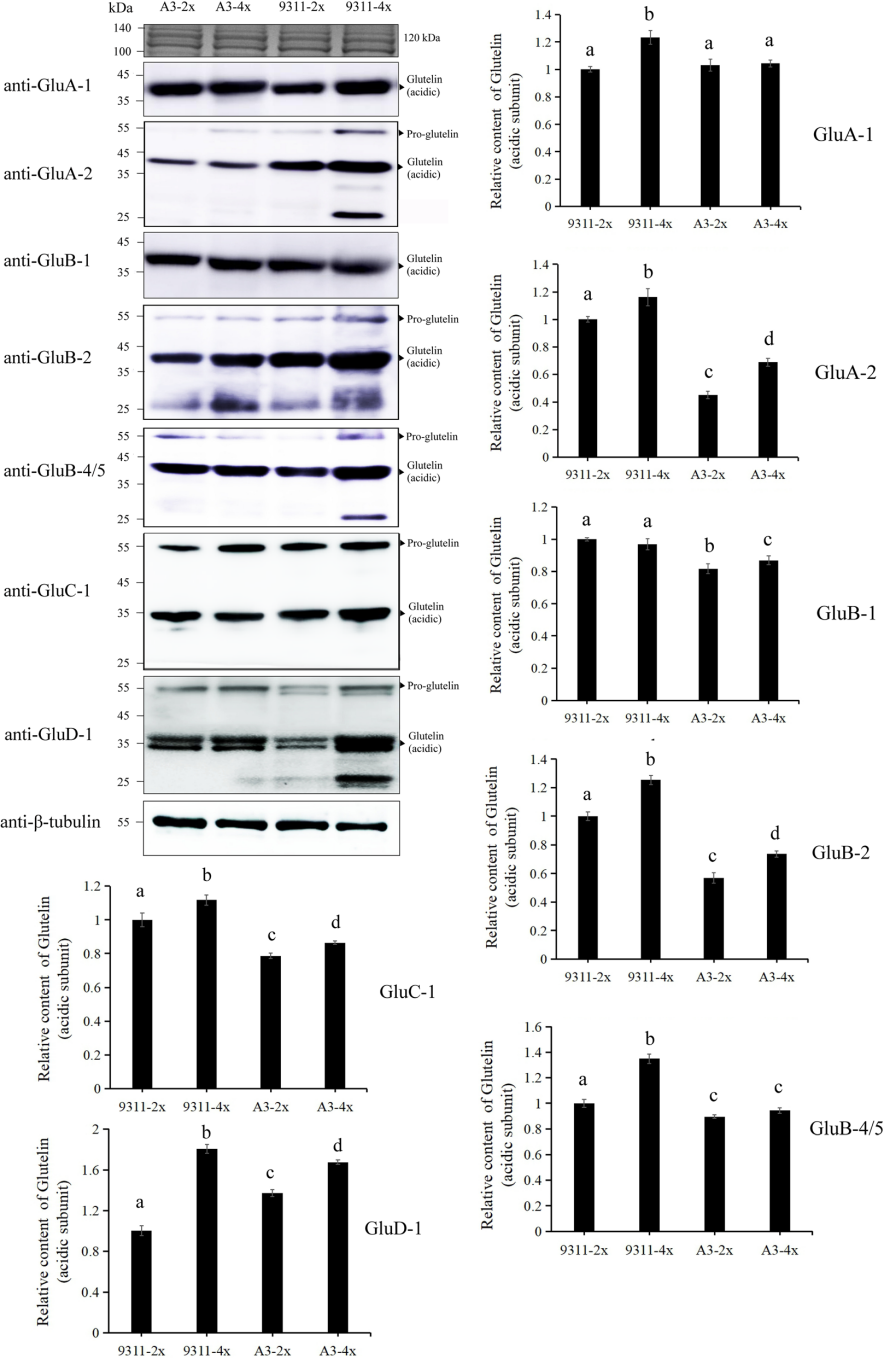
Immunoblot analyses of seven glutelin species. The protein extracts derived from mature rice seeds in 9311 and A3 (harvested in 2019) were separated by SDS-PAGE (4%–20% gradient), and analyzed by immunoblotting using anti-GluA-1, anti-GluA-2, anti-GluB-1, anti-GluB-2, anti-GluB-4/5, anti-GluC-1, and anti-GluD-1 antibodies. Immune bands of glutelin acidic subunit were scanned and quantified using Image J software. Different lowercase letters indicate significant differences (*p* < 0.05) as determined by one-way ANOVA and Tukey’s test. Each lane contained rice powder of similar weight. Black triangles represent pro-glutelin and glutelin acidic subunit. β-tubulin was used as a loading control. Three independent experiments were performed and data are shown

Analyses of protein bands revealed that three bands of approximately 57-kDa, 37-kDa and 20-kDa on the SDS-PAGE gel exhibited remarkable increases in tetraploid rice when compared with corresponding diploid rice (Fig. 1A and B). Variation in total protein content was primarily attributed to the increase in glutelin. Quantitative protein analyses based on TMT were independently performed three times using mature seeds to identify glutelins that were differentially expressed. Eight differentially expressed glutelins (≥1.5-fold) identified between 9311-2x and 9311-4x are presented in Table 1 (dataset S1). Glutelins in 9311-4x that exhibited increased expressions were identified as Glutelin type-A1 (GluA-1, Os01g0762500), Glutelin type-A2 (GluA-2, Os10g0400200), Glutelin type-A3 (GluA-3, OS03g0427300), Glutelin type-B2 (GluB-2, Os02g0249600), Glutelin type-B4 (GluB-4, Os02g0268300), Glutelin type-B5 (GluB-5, Os02g0242600), and Glutelin type-D1 (GluD-1, Os02g0249000). Furthermore, immunoblot bands of two pairs of cultivated varieties (9311-2x and 9311-4x, and A3-2x and A3-4x) were analyzed and the glutelin acidic subunits were quantified using Image J software. The results revealed that six glutelin gene bands (*GluA-1, GluA-2, GluB-2, GluB-4/5, GluC-1*, and *GluD-1*) increased in 9311-4x (Fig. 2), which was consistent with the results presented in Table 1. The signal intensities for anti-GluA-1, anti-GluA-2, anti-GluB-2, anti-GluB-4/5, and anti-GluC-1 antibodies were 1.1–1.3-fold higher in 9311-4x seeds than in 9311-2x seeds. The variation in the immune signal for anti-GluD-1 between 9311-2x and 9311-4x was the most significant, up to 1.8-fold (Fig. 2). In addition, the glutelin precursor was significantly up-regulated in diploid rice seeds relative to tetraploid rice seeds. Similar observations were made for immunoblot analysis results of A3-2x and A3-4x mature seeds. Glutelin expression levels regulated by *GluA-2*, *GluB-1*, *GluB-2*, *GluC-1*, and *GluD-1* were increased significantly when compared with glutelin expression levels in A3-2x, excluding *GluA-1* and *GluB-4/5* (Fig. 2). The variations in glutelin expression levels between 9311-2x and 9311-4x were more pronounced than variations in A3-2x and A3-4x, which verified the observation that the increase in glutelin in 9311 was considerably higher than that in A3 (Fig. 1A and B, Table S3).

**Table 1:** Identification of varying expression levels of glutelins and protein disulfide isomerase-like 1–1 (*PDIL1–1*) using tandem mass tags (≥1.5-fold) between 9311-2x and 9311-4x (harvested in 2019) at 25 days after pollination

Identified Protein	Coverage^a^ %	Theor:Mw(kDa)^b^	Peptides	Locus No.	Ratio	*P*-value	Gene name
Similar to Glutelin type-A1	49.5	56.239	27	Os01g0762500	1.700	0.000117	*GluA-1*
Glutelin type-A2	84.5	25.522	25	Os10g0400200	1.644	0.0000011	*GluA-2*
Glutelin type-A3	57.1	56.014	28	OS03g0427300	2.185	0.0000171	*GluA-3*
Similar to Glutelin type-B2	51.5	56.062	35	Os02g0249600	1.795	0.0000766	*GluB-2*
Similar to Glutelin type-B4	66.8	56.823	40	Os02g0268300	2.076	0.00402	*GluB-4*
GluB-5, glutelin precursor	40.7	56.835	27	Os02g0242600	2.417	0.0000007	*GluB-5*
Glutelin type-B5	37.3	54.693	25	Os02g0242600	1.654	0.000215	*GluB-7*
Similar to Glutelin type-B5	40.7	56.835	27	Os02g0242600	2.417	0.0000007	*GluB-7*
Glutelin type-D1	43.8	53.252	26	Os02g0249000	1.999	0.0000002	*GluD-1*
Protein disulfide isomerase-like 1–1	67.8	56.854	41	Os11g0199200	1.594	0.00000286	*PDIL1–1*

^a^Coverage denotes the percentage of protein sequence covered by the identified peptides

^b^theoretical molecular weight (MW)

### Effect of Polyploidization on the Dynamic Accumulation of Glutelins During Filling Stage

#### Dry and Fresh Weight Variations in Rice Seeds after Polyploidization

**Fig. 3:**
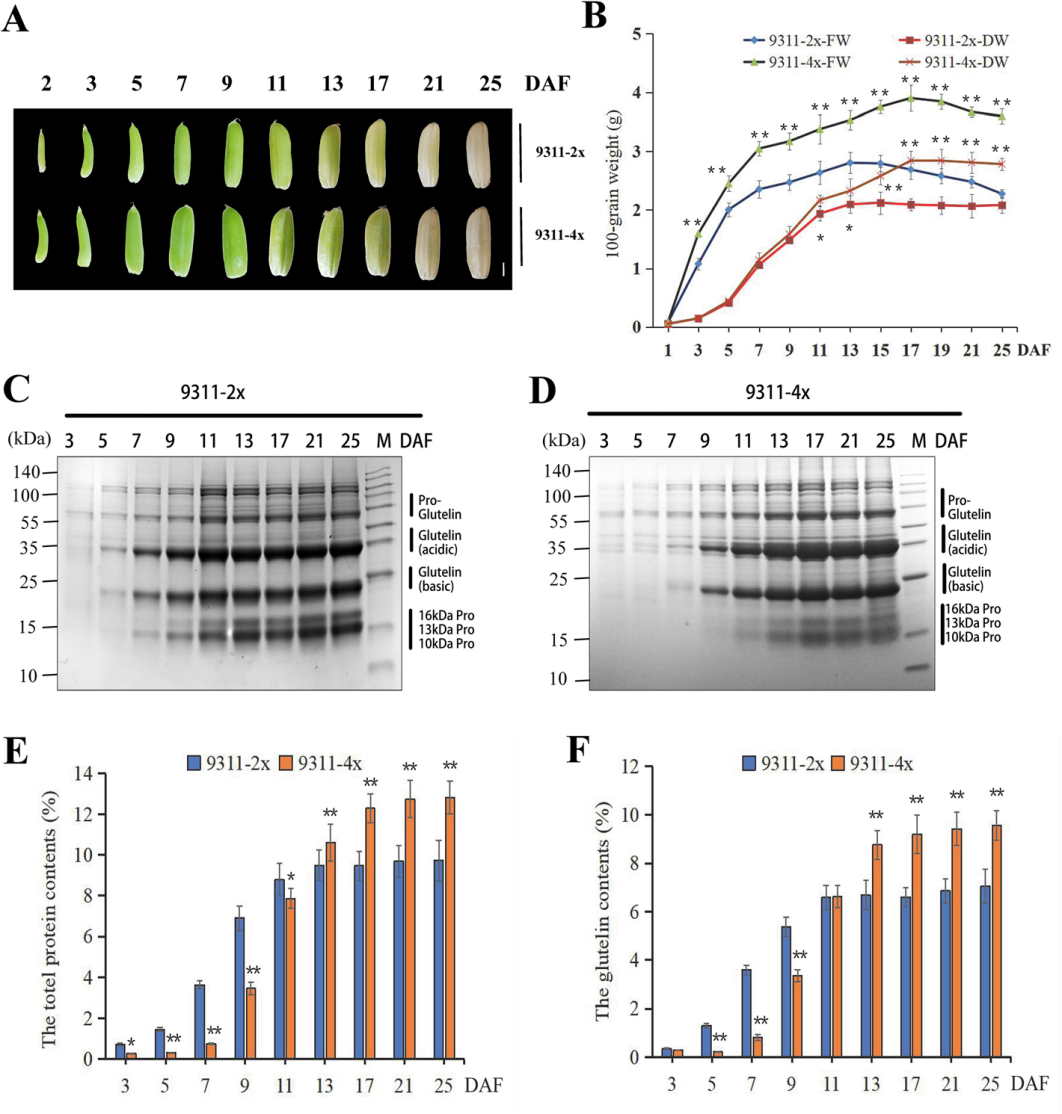
Variations in protein accumulation patterns in rice seeds with various ploidy levels during the filling stage. **A** Morphological changes between 9311-2x and 9311-4x rice seeds (harvested in 2019) from 2 to 25 days after flowering (DAF). Scale Bars: 1 mm. **B** Dry and fresh weights of 100 rice seeds from 3 to 25 DAF. One hundred seeds from the same part of three plants were considered one replicate. Three replicates were conducted. FW: fresh weight; DW: dry weight. Values are means ± SD. The asterisk indicates a statistically significant difference between 9311-2x and 9311-4x, as calculated by Student’s *t*-test (∗*p* < 0.05, ∗∗*p* < 0.01). **C**, **D** Coomassie brilliant blue (CBB) staining of total SSPs in immature seeds of 9311. Each lane contains rice seeds of similar weight. M = molecular size marker. Proteins were extracted from immature seeds at 3, 5, 7, 9, 11, 13, 17, 21, and 25 DAF. Pro-glutelin, glutelin acidic subunit, glutelin basic subunit, and prolamins (10, 13, and 16 kDa) are represented by black vertical lines. **E**, **F** Total seed protein and glutelin contents were analyzed using Image J software. Significant differences between tetraploid and diploid rice seeds were tested using independent Student’s *t*-test (∗*p* < 0.05, ∗∗*p* < 0.01). Data are presented as means ± standard errors of three biological replicates

WGD in the nucleus often results in certain morphological and physiological changes, such as leaf, fruit, flower, and seed enlargement. Polyploid rice exhibited a polyploid advantage in agronomic traits such as seed length, seed width, and 1000-seed weight, which were significantly higher than those in diploid rice (Song et al. 2007). The development process of 9311 seeds at the filling stage was monitored and we observed that certain indicators of tetraploid seeds, such as seed length, seed width, and fresh weight increased more rapidly than the indicators in diploid seeds (Fig. 3A and B). The fresh and dry weights of diploid seeds from 1 to 13 days after flowering (DAF) increased steadily and reached a maximum at 13 DAF (Fig. 3B). The fresh and dry weights did not vary with continued development, and fresh weight gradually decreased to a stable level (approximately equal to the dry weight) after water loss. Tetraploid rice seeds exhibited a similar trend, although the accumulation of organic matter took more days (Fig. 3A and B). Notably, no significant difference was observed in dry weights between diploid and tetraploid rice seeds during the early stage of seed filling (1–11 DAF; Fig. 3B). Although the volume of one tetraploid rice seed was larger, the dry weights of 100 seeds were approximately similar. We deduced that protein accumulation in diploid rice occurred preferentially, and the absolute organic matter content in a single diploid seed was higher than the content in a single tetraploid seed. After 13 DAF, the fresh and dry weights of tetraploid rice seeds increased significantly when compared with diploid seeds, which implied that the synthesis of diploid rice organs was inhibited, whereas that of tetraploid rice progressed.

#### Dynamic Accumulation of Glutelins During Filling Stages

The fresh weight, dry weight and total protein content of tetraploid rice seeds (9311-4x) increased by 41.39%, 31.36%, and 57.3%, respectively, when compared with those in mature seeds at 25 DAF in 9311-2x (Fig. 3B, Table S2). Total crude proteins of rice seeds (9311-2x and 9311-4x) collected between 3 and 25 DAF were extracted to investigate temporal expression patterns of glutelins. Protein samples were resolved by SDS-PAGE (4%–20%) gradient gels, stained using CBB, and analyzed using Image J software. Total protein and glutelin contents were faintly detected at 3 DAF and began to increase until approximately 11 DAF, then remained constant after 13 DAF in 9311-2x (Fig. 3C, E, and F). Comparatively, total protein and glutelins were synthesized at 3 DAF in 9311-4x, and their accumulation took 14 days to reach the maximum level, approximately 6 days after total protein and glutelin accumulated in 9311-2x (Fig. 3C–F). Rice glutelins contained 57-kDa glutelin precursors, and 37-kDa acidic and 20-kDa basic subunits (Tanaka et al. 1980; Yamagata and Tanaka 1986). Glutelin precursor protein was initially visible at approximately 3 DAF followed by the 37-kDa acidic subunit at 5 DAF and finally the 20-kDa basic subunit at 7 DAF in 9311-2x, as cleavage products of the precursor protein (Fig. 3C). Marked variations were observed in the expressions of the 37-kDa acidic and 20-kDa basic subunits in 9311-4x, which began manifesting at 7 DAF and were sustained until 17 DAF, and the subunit expression levels increased steadily as the seeds matured (Fig. 3D). In conclusion, the expressions of glutelin and its components in tetraploid rice seeds were delayed by 2 days and lasted 6 days longer than in diploid rice seeds.

### Effect of Polyploidization on Temporal Expressions of Various Genes Relevant to Glutelin Synthesis During Seed Filling Stage

**Fig. 4:**
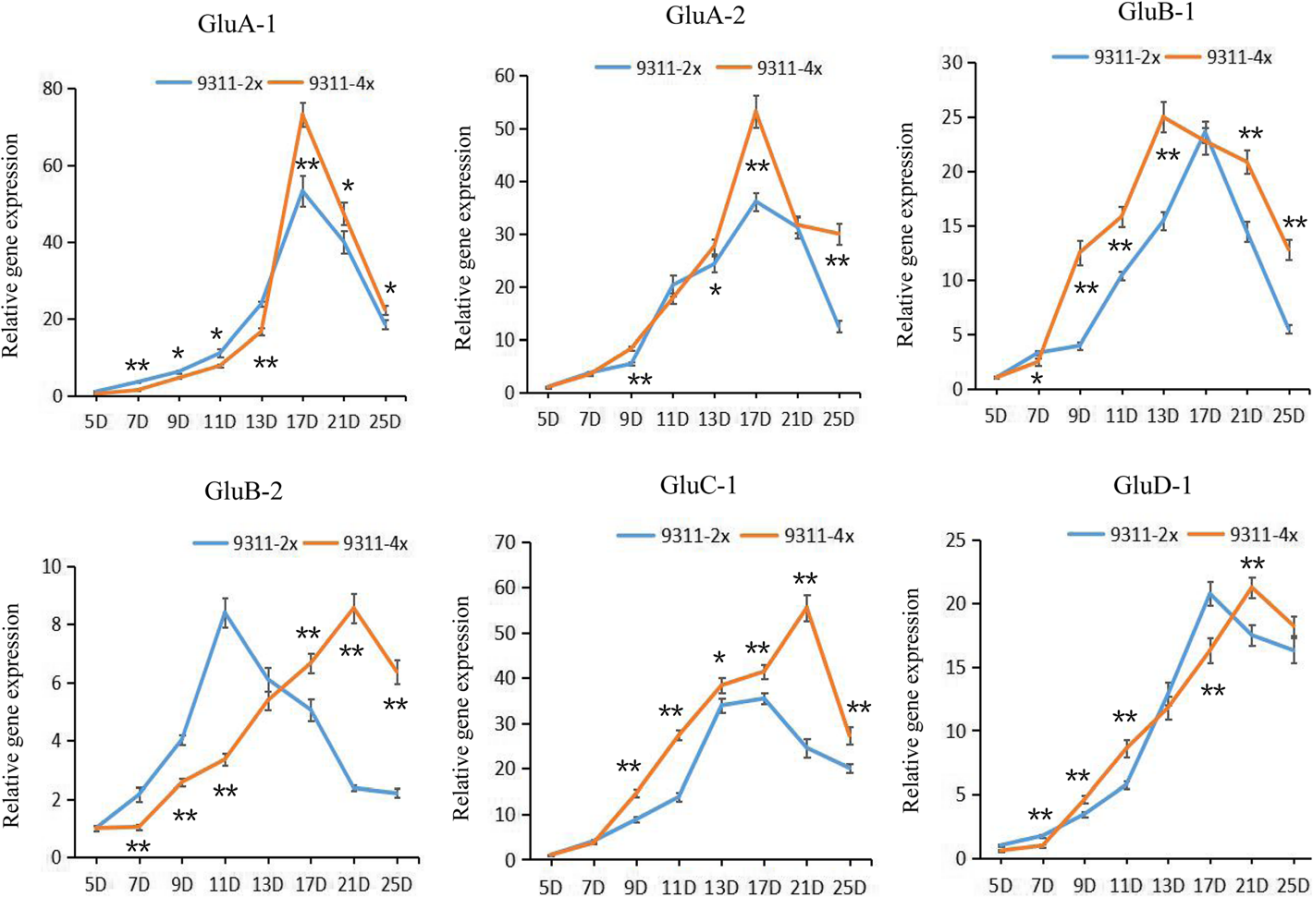
Variations in temporal expression patterns of various glutelin mRNAs in 9311-2x and 9311-4x (harvested in 2019). qRT-PCR analyses of glutelin gene expressions (*GluA-1*, *GluA-2*, *GluB-1*, *GluB-2*, *GluC-1*, and *GluD-1*) in developing seeds of 9311-2x and 9311-4x. The y-axis represents glutelin mRNA expression level relative to the β-actin mRNA level. The x-axis represents the day of seed collection after flowering. Significant differences in glutelin gene transcript levels between 9311-2x and 9311-4x at the same developmental stage were tested using independent Student’s *t*-test (∗*p* < 0.05, ∗∗*p* < 0.01). Data are presented as means ± standard errors of three biological replicates

A combination of SDS-PAGE and immunoblot analyses along with quantitative protein analyses revealed that glutelin content in tetraploid rice seeds increased considerably. Furthermore, to detect the expressions of the relevant glutelin genes, quantitative real-time PCR (qRT-PCR) analysis was performed using total RNA extracted from developing seeds at 5, 7, 9, 11, 13, 17, 21, and 25 DAF in 9311-2x and 9311-4x. Studies have reported that transcriptional expressions of rice glutelin genes in diploid rice were activated at 4–6 DAF, reached a maximum level at 10–14 DAF, and subsequently decreased (Krishnan and Okita 1986; Takaiwa and Oono 1991). In the present study, the expression levels of most glutelin gene mRNAs began to increase at 5 DAF, reached a maximum level at 17 DAF, and subsequently declined from 21 DAF in 9311-2x (Fig. 4). By contrast, *GluB-2* transcripts exhibited the highest levels of expression at 11 DAF. The expression trends of glutelin genes in 9311-4x were similar to those of 9311-2x, in which expression levels initially increased and subsequently decreased. However, various glutelin mRNAs in tetraploid rice still exhibited varying temporal expression patterns. The expressions of eight glutelin genes varied at different filling stages in 9311-4x when compared with 9311-2x. Most glutelin genes were expressed at significantly higher levels in 9311-4x than in 9311-2x during the late grain filling stage (after 17 DAF). Moreover, the expression peaks of three glutelin genes (*GluB-2*, *GluC-1,* and *GluD-1*) in 9311-4x were delayed to 21 DAF (4 days later), which demonstrated that glutelin mRNA expression in 9311-2x preceded expression in 9311-4x (Fig. 4). The results revealed that certain glutelin mRNA expressions in tetraploid rice were up-regulated during specific periods, and the expressions took longer to reach a peak than in diploid rice seeds.

### Effect of Polyploidization on Accumulation Patterns of Glutelin Polypeptides in Developing Seeds

**Fig. 5:**
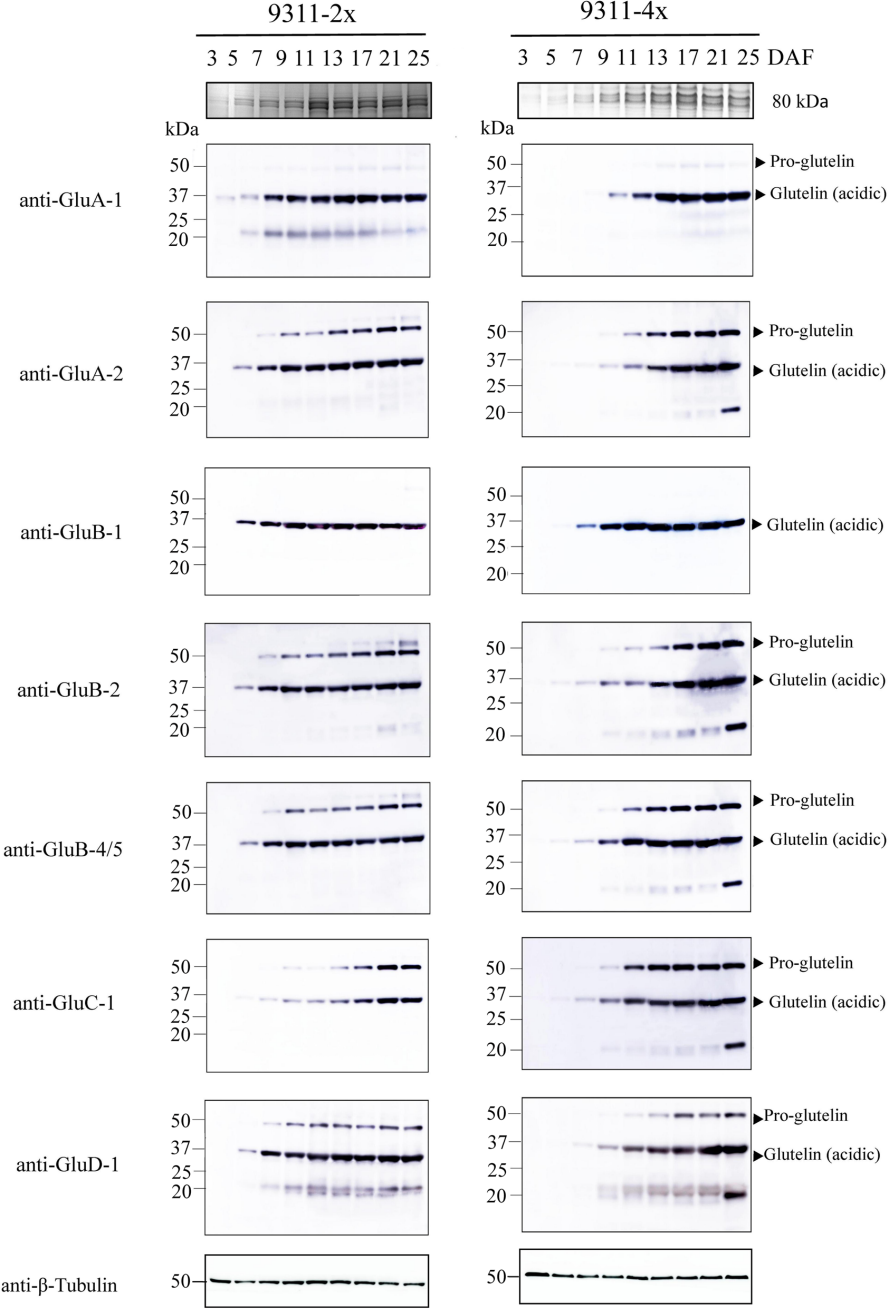
Accumulation patterns of various glutelin polypeptides in rice with varying ploidy levels. Total proteins in rice seeds from 3, 5, 7, 9, 11, 13, 17, 21, and 25 DAF in 9311-2x and 9311-4x (harvested in 2019) were subjected to immunoblotting analysis with anti-GluA-1, anti-GluA-2, anti-GluB-1, anti-GluB-2, anti-GluB-4/5, anti-GluC-1, and anti-GluD-1 antibodies. Each lane consists of rice seeds with similar weights. Pro-glutelins and glutelin acidic subunits are indicated by black arrowheads. β-tubulin was used as a loading control. Three independent experiments were performed and data are shown

Immunoblot analyses of each glutelin subfamily revealed the expression patterns of glutelins in 9311-2x and 9311-4x (Fig. 5). Similar glutelin subunits in rice with various ploidy levels were expressed at different stages, and the accumulation rates of various subunits varied. The 37-kDa acidic subunits synthesized by most glutelin genes were detected from 3 to 5 DAF, and the maximum glutelin expression level was reached at 9 to 11 DAF in 9311-2x, while the subunits were detected from 5 to 7 DAF, and glutelin expression levels started to peak at 13 to 17 DAF in 9311-4x, which is consistent with previous findings (Figs. 3C and D, 5); that is, 37-kDa acidic subunit accumulation in 9311-2x took 4–6 days and 6–16 days in 9311-4x. Contrary to expectations, the maximum glutelin expression levels regulated by *GluC-1* were attained at 21 DAF in 9311-2x, which occurred after the other gene expression levels had peaked, while the maximum glutelin expression levels regulated by *GluC-1* were attained at 13 DAF in 9311-4x, which lasted a shorter period of time than that in 9311-2x (Fig. 5). Overall, the present study demonstrated that the expression of the 37-kDa acidic subunit in tetraploid rice started a few days later and lasted longer.

Immunoblot analyses using anti-GluA-2, anti-GluB-2, anti-GluB-4/5, anti-GluC-1, and anti-GluD-1glutelin antibodies revealed that a 57-kDa glutelin precursor was also expressed. However, the starting time and duration of 57-kDa glutelin precursor expression were significantly different between diploid and tetraploid rice. The 57-kDa glutelin precursor was associated with four genes (*GluA-2, GluB-2, GluB-4/5*, and *GluC-1*) expressed at 7 DAF and its expression was sustained until 21 DAF in 9311-2x, while in 9311-4x, the precursor was expressed at 9 DAF and the expression sustained until 21 DAF or earlier; that is, 57-kDa glutelin precursor accumulation took 14 days in 9311-2x and 12 days in 9311-4x (Fig. 5). The results suggest that the expression of the 57-kDa glutelin precursor in tetraploid rice begins late and lasts a relatively short period, which differs from the 37-kDa acidic subunit accumulation patterns. Notably, contrary to other genes, 57-kDa glutelin precursor expression regulated by *GluD-1* reached a peak at 17 DAF in 9311-4x, which was considerably longer than the duration of peak expression in 9311-2x. Based on the results, we subsequently investigated the cytological accumulation mechanisms in immature seeds collected at 17 DAF and mature seeds collected at 25 DAF using SEM and TEM.

### Polyploid Rice Exhibits an Increased Thickness of Aleurone Cell Layers

**Fig. 6:**
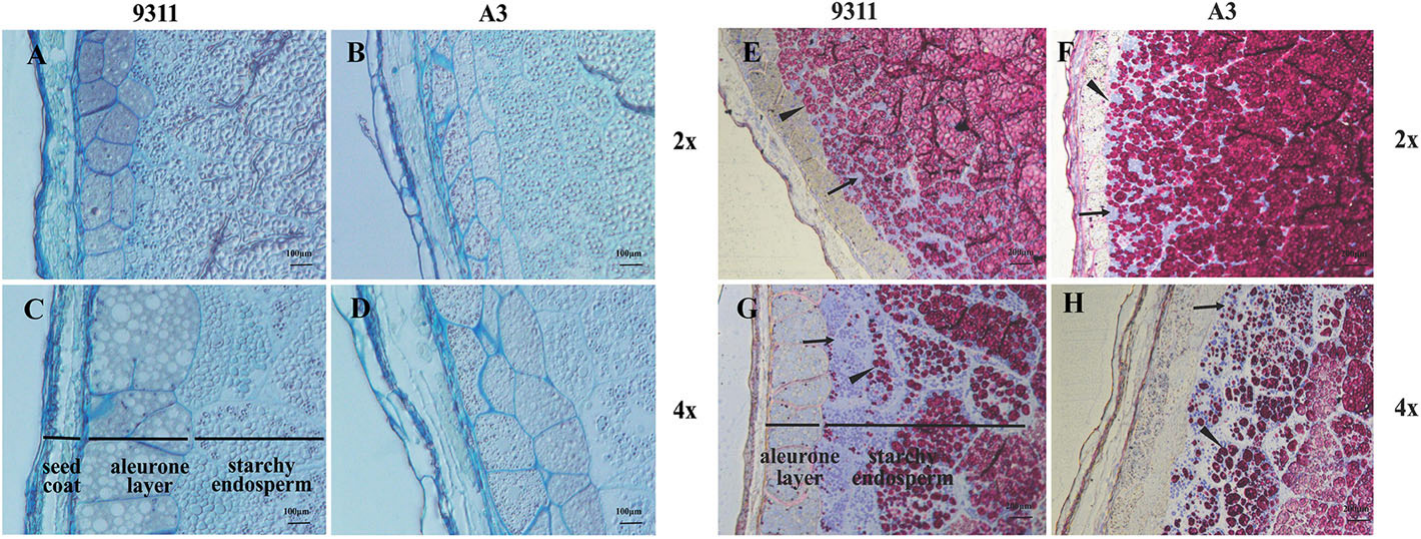
Variations in endosperm structural characterization in rice seeds with various ploidy levels. Semi-thin sections of the dehusked immature seeds of 9311 and A3 (harvested in 2018) at 17 DAF stained with methylene blue, (**A**–**D**) PAS, and CBB (**E**–**H**). Scale bar: 100 μm (**A**–**D**) and 200 μm (**E**–**H**). Seed coat, aleurone layer, and starchy endosperm are represented by black horizontal lines. Blue granules are indicated by black arrowheads, which represent plant proteins. Red granules are indicated by black triangles, which represent plant polysaccharides

**Fig. 7:**
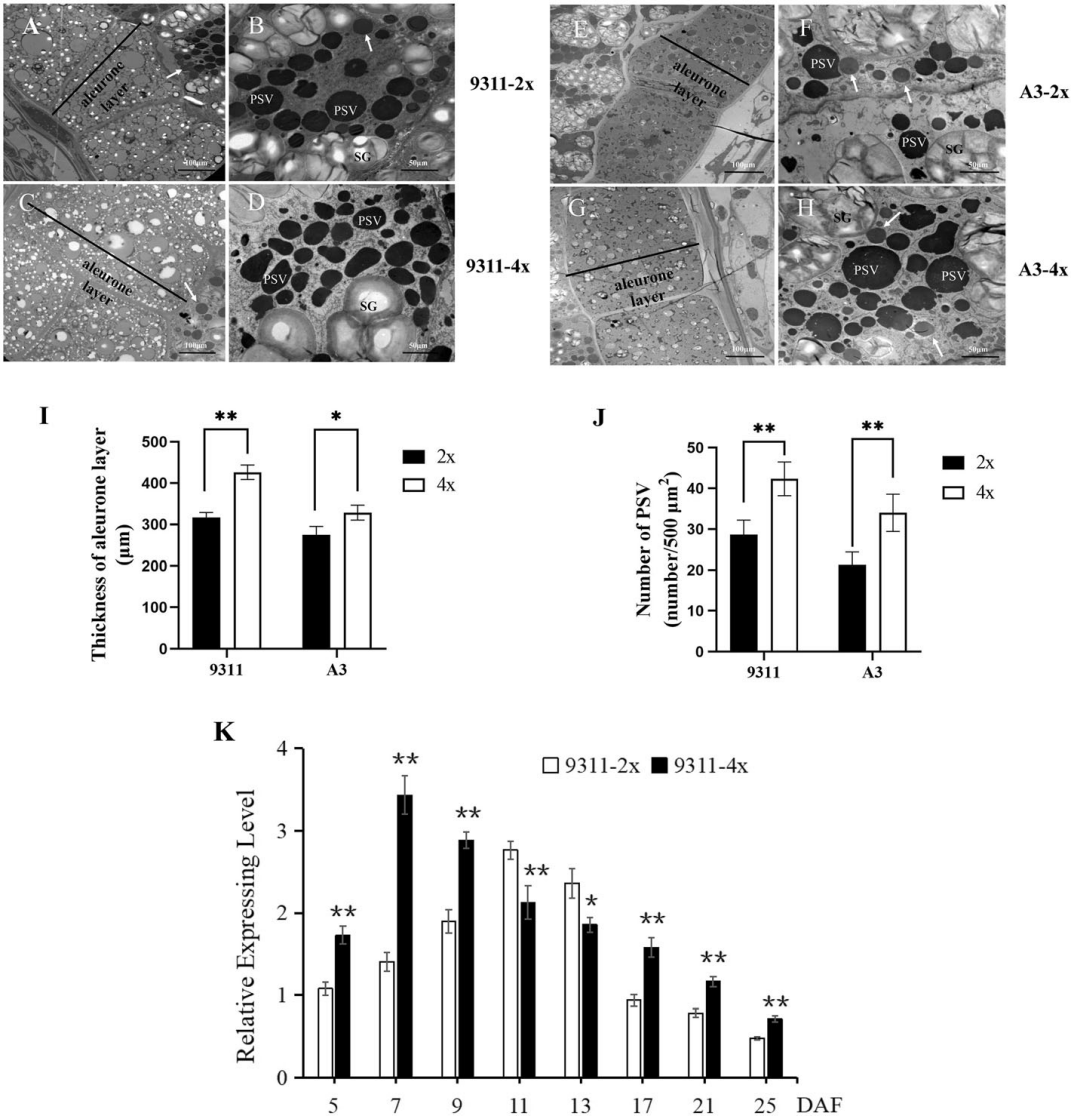
Variations in structural characteristics of PSVs in rice seeds with various ploidy levels. Electron microscopy of sub-aleurone cells in 9311 (**A**–**D**) and A3 (**E**–**H**) developing seeds at 17 DAF. **I** Thickness of aleurone layer. **J** Numbers of PSVs per 500 μm^2^. Data are presented as means ± standard errors of three biological replicates. SG-starch granule; PSV-protein storage vacuole; PB-I-Protein body-I. Significant differences between means of aleurone layer thickness and numbers of PSVs in tetraploid and diploid rice seeds were tested using independent Student’s *t*-test (∗*p* < 0.05, ∗∗*p* < 0.01). Scale bar: 50 μm. Arrows indicate protein body-I. **K** Temporal expression patterns of *PDIL1–1*. qRT-PCR analysis of the *PDIL1–1* expression level in developing seed endosperms of 9311-2x and 9311-4x (harvested in 2019). The y-axis represents mRNA expression level relative to the *β-Actin* mRNA level. The x-axis represents day of seed collection after flowering. Significant differences in *PDIL* transcript levels between 9311-2x and 9311-4x at the same developmental stage were tested using independent Student’s *t*-test (∗*p* < 0.05, ∗∗*p* < 0.01). Values are presented as means ± standard errors (*n* = 3 biological replicates)

A previous study revealed that aleurone layer thickness was positively correlated with an increase in SSP content (Wu et al. 2016). To identify polyploid endosperm phenotypes, seed endosperms of diploid and tetraploid rice were transversely sectioned. Afterward, cytohistological analyses were performed on semi-thin sectioned rice seeds that were stained with methylene blue (Fig. 6A–D), periodic acid–Schiff (PAS) reagent, and CBB (Fig. 6E–H). We examined the aleurone layer under LM, which revealed a significantly thicker aleurone layer in tetraploid rice seeds than in diploid rice seeds; however, the number of aleurone cell layers remained unaltered (Fig. 6A–D), and the observation was verified with SEM (Fig. S3) and TEM (Fig. 7A, C, E, and G) of seed sections at 17 and 25 DAF. Furthermore, aleurone layer thickness was evaluated by TEM. Results revealed that aleurone layer thickness in 9311-4x and A3-4x rice seeds increased by 32.92% and 23.64%, respectively, when compared with corresponding diploid rice seeds (Fig. 7I), which suggest that WGD influenced the development of the aleurone layer.

The starchy endosperm of diploid and tetraploid rice seeds was recognizable in transversally sectioned grains stained with PAS, which stained starch granules red, and CBB, which stained proteins blue. In contrast to diploid seeds (9311-2x and A3-2x), the starchy endosperm of tetraploid rice seeds (9311-4x and A3-4x) contained more densely packed protein granules and scattered starch granules (Fig. 6E–H), which increased protein content by 37.21% (9311-4x) and 22.30% (A3-4x), and decreased amylose content by 11.80% (9311-4x) and 16.15% (A3-4x) (Tables S2 and S5). Polyploidy is a key method of genetic improvement, which not only influences aleurone layer thickness, but also enhances protein accumulation in starchy endosperms.

### Alteration of PSV Structure in Tetraploid Rice Influences Glutelin Content

Subcellular structures in the sub-aleurone layer of endosperm cells were examined under TEM to elucidate the effect of WGD on PSV formation. PSVs were more prevalent than PB-Is in the sub-aleurone layer cells of diploid and tetraploid rice (Fig. 7B, D, F, and H). PB-I is spherical and has a concentric ring structure that is surrounded by rough ER membranes with attached polysomes (Saito et al. 2012). By contrast, PSV is an irregularly shaped granule with no lamellar structure, and is stained homogeneously (Fig. 7B, D, F, and H). In contrast to diploid rice (9311-2x, A3-2x), PSVs in tetraploid rice (9311-4x, A3-4x) were more irregular with a high frequency of occurrence, which increased by 47.67% and 59.37%, respectively (Fig. 7B, D, F, H, and J). The sub-aleurone layer of tetraploid rice seeds stained with PAS reagent and CBB appeared as blue granules under LM, which indicated more protein components (Fig. 6E–H). The findings suggest that WGD facilitates the formation of more PSVs, which, in turn, increases glutelin content.

### Effect of Polyploidization on the Expression Pattern of *PDIL1–1*

PDIL is a chaperone protein that catalyzes the formation of disulfide bonds in polypeptide chains, and is involved in oxidative protein folding by acting as a catalyst and facilitating folding activities in the ER (Muntz 1998; Kim et al. 2012a). In the present study, proteomic analyses of rice seeds at 25 DAF revealed that the expression of *PDIL1–1* in tetraploid rice increased by approximately 1.594-fold relative to the corresponding diploid rice (Table 1). SDS-PAGE analyses results also revealed that 57-kDa proglutelin, 37-kDa acidic, and 20-kDa basic subunits of tetraploid rice seeds improved significantly at 25 DAF (Fig. 1A and B). Furthermore, the expression of *PDIL1–1* and microstructures of PSVs at 25 DAF exhibited a similar trend. During the filling stage of tetraploid rice seeds, *PDIL1–1* expression reached a peak at 7 DAF (4 days earlier than diploid rice seeds), which was higher than that of diploid rice seeds, except at 11–13 DAF (Fig. 7K). However, according to a previous study, the expression of *PDIL1–1* in diploid rice increased gradually after flowering, peaked at 11 DAF, and subsequently decreased sharply to basal levels (Kim et al. 2012a). The results of the present study revealed that tetraploid rice exhibited varying temporal expression patterns of *PDIL1–1*, and a positive correlation was observed between *PDIL1–1* expression and the number of PSVs, which could promote glutelin accumulation in tetraploid rice. We speculated that increased glutelin synthesis requires a higher expression of *PDIL1–1* to enhance processing and folding of glutelin, in turn, increasing glutelin accumulation.

## Discussion

### Polyploidy is a Potential Approach of Increasing SSPs

Polyploidy exhibits substantial enhancement potential and high adaptability, such as robust growth, enhanced stress resistance, high biological yield, fruit enlargement, and nutrient content enhancement, when compared with diploidy (Yu et al. 2020). The actual existence of tetraploid plants in nature could be demonstrated by increasing studies despite the potential limitations (Soltis and Soltis 2009; Parisod et al. 2010). WGD increases gene dosage, genetic reservoirs, and combinatorial complexity, which, in turn, enhance the evolutionary success of polyploidy in plants (Jiao et al. 2011; Madlung and Wendel 2013; Xiong et al. 2019).

A few studies have revealed that nutritional enhancement in food crops is a fundamental goal in modern agriculture that is achievable by increasing the contents of proteins, amylose, amino acids, vitamins, minerals, or dietary fibers to satisfy individual nutritional requirements (Sun and Liu 2004; Pfeiffer and McClafferty 2007). The dosages and structures of hereditary substances in tetraploids have been modified due to the influence of doubling and non-doubling factors involved in the process of WGD when compared with corresponding diploids, which, in turn, lead to variation in tetraploid-related traits. Some progress has been made in annual cereal crop breeding, such as the development of autotetraploid rye, wheat, sorghum, and rice; the contents of carbohydrates, proteins, vitamins, and alkaloids in some autopolyploid plants are higher than the contents in corresponding diploid plants (Tiwari and Xu 1982; Comai 2005; Cai et al. 2007). A previous study revealed that the absolute contents of glutelin and albumin increased significantly after polyploidization, while the absolute contents of gliadin and globulin decreased slightly, resulting in an increase in total protein content (Tiwari and Xu 1982).

Rice seeds are deficient in certain essential amino acids, which leads to the imbalance in amino acid content. Protein content and amino acid composition are crucial factors that determine the nutritional quality and usability for producers and consumers (Kim et al. 2012b). The results of the present study have demonstrated the feasibility of enhancing the general nutritional profile of rice by doubling rice chromosomes. Total brown rice protein, glutelin, and amino acid contents in tetraploid rice seeds were considerably enhanced when compared with corresponding diploid rice seeds. The increase in total protein content was primarily attributed to the increase in glutelin and prolamins, with the exception of albumin and globulin contents (Fig. 1). The nutritional value of rice glutelin is higher than the nutritional value of prolamin, albumin and globulin, which are largely indicated by the high amounts of essential amino acids, especially the first limiting amino acid (lysine). The nutritional value of rice can be increased considerably by enhancing lysine content and utilization of total proteins in the seeds. WGD can enhance the nutritional quality of rice by increasing lysine content. In addition, the synthesis of specific new proteins in tetraploid rice seeds after polyploidization has not been studied. Only one polyploid variety (9311-4x) exhibited a distinct non-specific band (Figs. 2 and 5) among the varieties studied. Similar non-specific bands have been observed in previous studies using similar glutelin antibodies in diploid rice (Takahashi et al. 2019). We speculated that gene dosage and multiple gene interactions in polyploid rice could result in a new specific band, which is homologous with the acid subunit.

### Effect of Polyploidization on Glutelin Biosynthesis, Transport, and Deposition

Previous researchers have demonstrated that polyploid rice exhibits considerable potential value, and tetraploid rice could be a key germplasm in studies using polyploidy to enhance rice yields (Cai et al. 2007; Song et al. 2007). Most glutelin genes or cDNAs have been cloned in diploid rice and although the expression and regulation of glutelin genes (Table S1), and the cellular processes underlying glutelin biosynthesis, transport, and deposition have been elucidated, relatively less research has focused on tetraploid rice due to lack of special tetraploid rice lines. Based on previous studies on diploid rice, the present study has presented a more comprehensive breakthrough with regard to the exploration of the variable expression and synthesis of glutelins, and the histological characteristics of the endosperm in tetraploid rice. Synthesis of glutelin requires a series of complex physiological and biochemical metabolic processes (Kim et al. 2013). The genes coding for glutelin in tetraploid rice are identical to those in diploid rice, and the main variation exists in the expression patterns including spatio-temporal patterns. The increase in gene dosage and multiple gene interactions could result in the overexpression of certain glutelins, which was one of the key molecular events involved in protein constitution or content modifications in rice endosperms. A comparison of mRNA and protein expressions of glutelins between tetraploid and diploid rice at different developmental stages revealed that polyploidization not only altered glutelin gene expression levels at specific filling stages, but also caused a delay in the initiation and prolongation of glutelin synthesis, which resulted in an increase in the accumulation of glutelins (Figs. 4 and 5). The protein content of tetraploid barley seeds induced by colchicine increased by 52%, and continuous evaluation of seeds over several generations revealed that the increase was stable and reliable (Tiwari and Xu 1982). Therefore, the results could be closely associated with the delay in the growth period and accumulation of more proteins in tetraploid rice.

SSPs synthesized during seed maturation are deposited into the PBs from ER lumen via a process mediated by ER chaperones (Kumamaru et al. 2010). Glutelin precursors are synthesized in membrane-bound ribosomes, transported to the ER cavity, folded and assembled with the assistance of molecular chaperones in the ER to form disulfide bonds in peptide chains (Krishnan et al. 1992; Kumamaru et al. 2010). The loss-of-function mutants of ER chaperones resulted in a decrease in protein contents and phenotypic variations in starch granules in rice seed endosperms (Li et al. 1993; Muench et al. 1997; Yasuda et al. 2009; Onda et al. 2011). Binding protein (BiP) interacts with immature proteins on the ER lumen, which facilitates protein folding (Yasuda et al. 2009). Calnexin (CNX) selectively binds to the unfolded glycoproteins, which can prevent transportation of misfolded proteins from the ER to the Golgi apparatus (Kleizen and Braakman 2004). PDI, a catalyst of disulfide bond formation and rearrangement, is also a molecular chaperone that facilitates polypeptide folding and is a key factor influencing storage protein biogenesis (Bulleid and Freedman 1988). In PDI deficient mutants, disulfide bonds in the peptide bond of glutelin precursor could not be synthesized and processed normally, resulting in a substantial increase in glutelin precursor and a significant decrease in mature glutelin acidic and basic subunits (Takemoto et al. 2002). No significant difference was observed in the expressions of BiP and CNX based on proteomics results; however, the expression of *PDIL1–1*, which facilitates oxidative folding of vacuole-targeted storage proteins, such as proglutelins and α-globulin (Kim et al. 2012a), exhibited marked variations between 9311-2x and 9311-4x rice seeds at 25 DAF. Proglutelins (Fig. 1A and B) and α-globulin (data not shown) in the 9311-4x rice seeds at 25 DAF increased significantly, which implies that ER chaperones, such as *PDIL1–1*, were highly expressed at the transcript and protein levels (Table 1; Fig. 7k).

Numerous studies have revealed that the thickness and number of aleurone cell layers are positively correlated with rice seed protein content (Kawakatsu et al. 2009; Liu et al. 2018). We have demonstrated that tetraploid rice exhibits increased aleurone cell layer thickness, and an enhanced glutelin content and nutritional profile. Furthermore, glutelin biosynthesis, transport, and deposition in tetraploid rice, which determine the nutritional quality of rice seeds, were investigated. Recent studies have focused on the folding and sorting mechanisms of glutelins and prolamins. Enhancement of glutelin can be achieved by interfering with the expression of other storage proteins, coupled with alterations in the shape and size of the PBs (Kim et al. 2013; Lee et al. 2015). After polyploidization, the contents of three protein components (glutelins, prolamins, and globulins) increased to varying degrees (Fig. 1A; Table S3). Therefore, we inferred that glutelin alterations in tetraploid rice may not be associated with compensatory accumulation due to inhibition of the expression of one protein component. RNA-binding proteins specifically bind to glutelin mRNA sequences and regulate glutelin mRNA localization, and the partial loss of its function mislocalizes glutelin and prolamin mRNAs (Tian et al. 2018). Mislocalization of α-globulin RNA to the cisternal ER disrupts transportation of glutelin to the PSVs and their packaging (Yang et al. 2014). In addition, PSVs exhibited varied morphology and increased quantity with continuous accumulation of glutelin and globulin in tetraploid rice, suggesting that glutelin deposition was normal and there was no mislocalization.

### Morphological Variations in Starch Granules of Seed Endosperms of Tetraploid Rice

Protein and starch contents are two key indices considered in the evaluation of the nutritional value of rice. Starch, which is composed of amylose and amylopectin, is a key component of the rice endosperm. The proportions of endosperm contents determine the eating and cooking qualities of rice, and appropriate amylose content is a key indicator of high-quality rice (Pang et al. 2016). Starch granule morphology is associated with the quality of rice. Starch granules in rice seeds with superior quality are smaller, with a distinct polyhedral crystal shape, clear and visible edges and corners, neat and compact arrangement, and a small seed gap (Ji et al. 1998; Cho et al. 2016). Conversely, the starch granules of poor quality seeds and in chalky parts of the rice seed are not uniform in size; the polyhedral structure is not well-defined; and the granules are loose (Cho et al. 2016). The favorable traits in the production of tetraploid rice are high protein and amino acid contents, and low amylose content (Cai et al. 2007). In the present study, amylose contents of six pairs of rice varieties were evaluated and the results revealed that polyploidization significantly influenced amylose contents, which exhibited similar downward trends based on different genotypes (Table S5). In addition, sectioned specimens of diploid rice were lucid, whereas those of tetraploid rice were opaque with a floury feature (data not shown). The micro-structure of starch granules was observed using SEM and the results revealed that starch granules in seed endosperms of tetraploid rice (9311-4x and A3-4x) were more tightly packed, smaller in sizes and with more regular polyhedral shapes when compared with those in diploid rice (9311-2x and A3-2x) (Fig. S4). The morphological variations in starch granules could explain the opaque and floury features observed in tetraploid rice seeds, and could be a key factor influencing the decline in seed starch content of tetraploid rice. The observations could imply that polyploidization influences the balance between protein and amylose; a decrease in amylose content could be a factor contributing to an increase in total protein and glutelin content, which provides a new insight into the enhancement of nutritional quality of rice by polyploid breeding.

## Conclusions

WGD exerts a great effect on the biosynthesis and accumulation of glutelin in rice seeds. In the present study, total brown rice protein, glutelin, and amino acid contents in tetraploid and diploid rice seeds were systematically evaluated and the results suggested that tetraploid rice was more nutritious than diploid rice to a certain extent. TMT and immunoblot analyses were performed to identify eight differentially expressed glutelins between 9311-2x and 9311-4x. The effect of polyploidization on temporal expressions of various glutelin genes was manifested in the initial time, duration, and relative levels during seed filling. The cytological factors of polyploidy influencing glutelin deposition primarily in the form of increase in aleurone layer thickness and PSVs were explored. Finally, increase in the biosynthesis of glutelin precursor in tetraploid rice after polyploidization could require production of high levels of PDIL as a chaperone to facilitate folding and localization of the protein.

## Materials and Methods

### Plant Materials and Growth Conditions

A total of 24 pairs of brown rice cultivars including tetraploid rice and the corresponding diploid rice cultivars were used for the experiments. The rice cultivars were grown at Hubei polyploid rice breeding base, and harvested between 2018 and 2019.

Autotetraploid rice lines (4n = 48) were artificially synthesized from *O. sativa* ssp. indica (2n = 24) and *O. sativa L. japonica* (2n = 24). Tetraploid plants with doubled genome were obtained by treating rice seed buds or callus with a 0.05% (wt/vol) aqueous solution of colchicine for 48 h at 28 °C, and carried out plant architecture screening and chromosome counting for the two generations. Over the last decade, our research group developed several tetraploid indica–japonica hybrid rice through artificial hybridization between tetraploid indica and japonica hybrid lines, and subsequently selected excellent hybrid lines by backcrossing or multiple crossing, then bred corresponding diploid rice using anther culture techniques. Diploid and tetraploid lines were self-pollinated over 48 generations with panicle bagging to prevent cross-pollination.

### Identification of Glutelin Genes

To identify the genes encoding rice glutelin, we performed sequence similarity searches using publicly available sequences of rice in the National Center for Biotechnology Information database (http://www.ncbi.nlm.nih.gov/) and the Rice Annotation Project database (http://rapdb.dna.affrc.go.jp/), and selected genes with high percentage identity using BLAST searches of rice (*O. sativa* L. cv. Nipponbare) genome. A search of the Rice Genome Annotation Project database (http://rice.plantbiology.msu.edu/) was performed using glutelin as a keyword to identify glutelin genes. Redundant sequences were removed by aligning the gene sequences and their accuracy was checked using the ID converter system in the Rice Annotation Project database.

### Determination of Nutritional Contents

Total proteins were quantified according to the NY/T3–1982 standard of crude protein content determination in cereals and legumes (semimicro-Kjeldahl method). Amylose contents were quantified according to a previously described method (Liu et al. 2018). A total of 17 amino acids were quantified (three replicates) using a previously described method (Kim et al. 2013) to determine amino acid content. Glutelins and prolamins were extracted based on the method of separating storage proteins in seed endosperms (Kumamaru et al. 1988). Prolamin contents were determined using Bradford Protein Assay Kit (Sigma-Aldrich, St. Louis, MO, USA). Glutelin content was determined using a Bicinchoninic Acid (BCA) Kit (Sigma-Aldrich). Amylose content was determined using an Amylose Assay Kit (Megazyme International Ireland Ltd., Wicklow, Ireland).

### Measurement of Fresh and Dry Weights at the Filling Stage

A total of 100 seeds at the filling stage from 1 DAF were collected after every few days (1, 3, 5, 7, 9, 11, 13, 17, 21, and 25 DAF), and subsequently manually shelled and mixed, and the fresh weight of seeds measured; each measurement was repeated three times. Afterward, the seeds were killed at 105 °C and dried at 80 °C until a constant weight was obtained, after which the dry was weight determined.

### Total Protein Extraction from Endosperm and Analysis by SDS-PAGE

In 2019, developing seeds of 9311 and A3 cultivars were sampled in the morning at 3, 5, 7, 9, 11, 13, 17, 21, and 25 DAF and immediately stripped of the hulls before being stored at − 80 °C until required.

Rice powder (100 mg fresh weight) was suspended in 1000 μL protein extraction buffer (8 M urea, 4% SDS, 250 mM Tris-HCl (pH 6.8), 20% glycerol, 5% ME, and 100 μg/mL PMSF) and total proteins extracted by shaking overnight at 37 °C. The aqueous supernatants were collected after centrifuging at 12,000×g for 20 min at room temperature, and quantified using Micro BCA assay reagent (Pierce; Thermo Scientific, Waltham, MA, USA). The proteins (50 μg) were denatured by boiling in water for 5–10 min and separated using 4–20% Tris-Glycine gels (Invitrogen, Carlsbad, CA, USA), and subsequently stained with 0.1% CBB R-250 and transferred to a polyvinylidene fluoride membrane for immunoblot analyses. The gel was scanned using an Amersham Imager 680 (Cytiva, Marlborough, MA, USA) and relative accumulation levels calculated using Image J software (http://rsbweb.nih.gov/ij/).

### Preparation of Polyclonal Antibodies and Immunoblot Analysis

With reference to a previous description, synthetic peptides were designed to produce seven types of polyclonal anti-glutelin antibodies (anti-GluA-1, anti-GluA-2, anti-GluB-1, anti-GluB-2, anti-GluB-4/5, anti-GluC-1, and anti-GLUD-1) based on variable regions (Kawakatsu et al. 2008; He et al. 2013; Takahashi et al. 2019) (Table S6). The antibodies were prepared from rabbits by injecting corresponding synthetic peptides (Bioconsumable Biotechnology Co., Ltd., Beijing, China). Afterward, we used the corresponding synthetic peptide conjugated columns to purify polyclonal antibodies from rabbit serum by affinity chromatography, and subsequently classified and stored the antibodies at − 80 °C until required. Anti-β-tubulin (AS10 681) antibody produced in rabbits was purchased from Agrisera antibodies (Vännäs, Sweden).

Developing seeds at 3, 5, 7, 9, 11, 13, 17, 21, and 25 DAF were sampled for immunoblot analyses as described previously (Kawakatsu et al. 2008). Relative accumulation levels of proteins were calculated from the immunoblot band intensities on X-ray films using Image J software (http://rsbweb.nih.gov/ij/).

### RNA Extraction and qRT-PCR

Total RNA was extracted from developing seeds (5, 7, 9, 11, 13, 17, 21, and 25 DAF) using a plant RNA extraction kit (TaKaRa, Dalian, China; https://www.takarabiomed.com.cn/) as a template. The first strand of cDNA was synthesized using a reverse transcription kit with olig (dT)-primer and then amplified by PCR (TaKaRa, Dalian, China). We designed several pairs of specific primers used for storage protein genes, ER-stress response genes, and internal reference genes (Actin and Ubiquitin) for qRT-PCR analyses (Table S7). qRT-PCR analyses were performed using SYBR Premix Ex Taq kit (TaKaRa, Dalian, China), a CFX real-time PCR system and system software (Bio-Rad, Hercules, CA, USA), according to the manufacturer’s instructions. Each seed RNA was extracted from at least three individual plants. Three independent groups of RNA samples were extracted from developing seeds collected at different periods for qRT-PCR analyses. Three technical replicates of each biological replicate were used for each sample. The melting curves at the end of each reaction were analyzed to ensure specificity of PCR products.

### Histological Analysis of Semi-Thin Sections

Husks were removed manually from developing rice seeds collected at 17 DAF and the seeds sectioned transversely into 12 mm slices, and fixed immediately in glutaraldehyde solution (2.5% glutaraldehyde, 0.1 M phosphate buffer [pH 7.3]) for 24 h. Dehydration, embedding, and slicing of the samples were performed as described previously (Wu et al. 2016). Two staining methods were used to examine the structures of semi-thin sections (1.25 μm). First, the sections were stained with PAS reagent, and counter-stained with 0.1% (w/v) CBB for 15 min as described previously (Wu et al. 2016). In addition, the sections were dyed to a metallic color with Toluidine blue, washed three times with double distilled water and dried on a baking board. Photographs were taken using a Nikon microscope (Eclipse 80i; Nikon, Tokyo, Japan).

### SEM and TEM

Immature seeds at 17 DAF and mature seeds at 25 DAF from diploid and tetraploid rice were harvested and fixed overnight in 2.5% glutaraldehyde in 0.1 M phosphate buffer (pH 7.3) at 4 °C (Saito et al. 2012). SEM and TEM were performed as described previously (Wang et al. 2010; Saito et al. 2012).

### Proteomic Analysis

Harvested mature seeds were immediately stored at − 80 °C until required. Three biological replicates of mature seeds from 9311-2x and 9311-4x were pooled for TMT analyses. Rice seeds were ground into fine powder in liquid nitrogen. Protein extraction was performed in a lysis buffer (Roche) according to the manufacturer’s instructions and stored overnight at − 20 °C. Protein concentration was determined using an enhanced BCA Protein Assay Kit (P0010; Beyotime Biotechnologies, Ltd., Beijing, China) according to the manufacturer’s instructions. Each protein sample (200 μg) was digested with trypsin overnight at a trypsin-to-protein ratio of 1:100 and subsequently desalted by elution from a Strata-X C18 SPE column (Phenomenex, Torrance, CA, USA), and vacuum dried. The peptides were reconstituted in 0.5 M triethylammonium bicarbonate buffer, and each sample was labeled using 2-plex TMT kit (Frankfurt am Main, Germany) according to the method described in a previous study (Zhang et al. 2017). After labeling, individual TMT 2-plex samples were mixed and diluted into 0.1% trifluoricacetic acid, followed by loading on a MacroSpin Vydac C18 reverse phase mini-column (The Nest Group Inc., Southborough, MA, USA).

Liquid chromatography-tandem mass spectrometry (LC-MS/MS) analyses for TMT-labeled samples were performed using a Q Exactive™ Orbitrap mass spectrometer (Thermo Fisher Scientific, San Jose, CA, USA) coupled to an EASY-nLC 1000 (Thermo Fisher Scientific, San Jose, CA, USA). Peptide identification and quantification was performed by searching the LC-MS/MS spectra data against an assembly data file using the Mascot 2.2 and Proteome Discoverer™ 1.4 (Thermo Fisher Scientific, San Jose, CA, USA). A unique protein with at least two unique peptides that had a false discovery rate < 0.0160 was used for data analyses. Protein quantification was based on the total intensity of assigned peptides. An average of eight labeled sample mixes was used as a reference and was based on a weighted average of the intensity of reported ions in each peptide identified. Final protein ratios were normalized to the median average protein content of the 8-plex samples. Fold change values (FC) > 1.2 for upregulated or FC < 0.83 for downregulated proteins were set as the threshold for identifying differentially expressed proteins.

### Statistical Analysis

At least three biological replicates were used for the analyses of each treatment and control group. Amino acids, protein content, and fresh and dry weights were analyzed using MS Excel (Microsoft Corp., Redmond, WA, USA) and SPSS 17.0 (SPSS Inc., Chicago, IL, USA). Data were analyzed by one-way analysis of variance and the means were compared by least significant difference test at 5% probability level.

## Supplementary Information

**Additional file 1: Figure S1.** Phylogenic relationships among rice glutelin proteins. A rooted tree was generated based on a multiple sequence alignment using MEGA X. Glutelins were grouped into four distinct sub-clades (*GluA, GluB, GluC and GluD*). **Figure S2**. Expression analysis of SSPs using gradient SDS-PAGE (4%–20%). CBB staining of total protein in five pairs of mature rice seeds (NJ11-2x and NJ11-4x, CX35-2x and CX35-4x, Mudgo-2x and Mudgo-4x, HJK-2x and HJK-4x, and Balilla-2x and Balilla-4x, harvested in 2019). Each lane contains rice grains of same weight. M = molecular size marker. Proteins were extracted from rice seeds at 25 DAF. Pro-glutelin polypeptides, glutelin acidic and basic subunits, and prolamins are indicated by black vertical lines. **Figure S3**. Variations in aleurone layer structural characterization in rice seeds with various ploidy levels. SEM images of aleurone layers and endosperms of 9311-2x and 9311-4x seeds at 17 and 25 DAF (harvested in 2018). Scale bar: 50 μm. **Figure S4.** Variations in starchy endosperm structural characterization in rice seeds with various ploidy levels. SEM images of seed endosperms in two pairs of rice lines (9311-2x and 9311-4x, and A3-2x and A3-4x, harvested in 2018) at 25 DAF. Scale bar: 10 μm, 50 μm and 500 μm.

**Additional file 2: Table S1.** Classification of rice glutelin genes. **Table S2.** Total protein content and average growth of tetraploid brown rice seeds and the corresponding diploid rice seeds harvested in Wuhan between November 2018 and November 2019. Data are presented as means ± standard errors of three biological replicates; * denotes significant differences in protein content between tetraploid and diploid rice (*p* < 0.05); ** denotes extremely significant differences (*p* < 0.01). **Table S3.** Glutelin and prolamin contents in tetraploid and diploid rice seeds (harvested in 2019). * denotes significant differences in component protein content between tetraploid and diploid rice (*p* < 0.05); ** denotes extremely significant differences (*p* < 0.01). **Table S4. S**eventeen amino acid contents of tetraploid and the corresponding diploid brown rice seeds (harvested in 2019). Data are presented means ± standard errors of three biological replicates; * denotes significant differences in amino acid contents between tetraploid and the corresponding diploid rice (*p* < 0.05); ** denotes extremely significant differences (*p* < 0.01). **Table S5.** Amylose contents of tetraploid and diploid rice (harvested in 2019). Data are presented as means ± standard errors of three biological replicates. * denotes significant differences in amylose contents between tetraploid and diploid rice (*p* < 0.05). ** denotes extremely significant differences (*p* < 0.01). **Table S6.** Synthetic peptides for polyclonal anti-glutelin antibodies. **Table S7.** Primer sequences used for qRT-PCR.

**Additional file 3: Dataset S1.** The list of all proteins identified using LC-MS/MS in 9311-4x and 9311-2x (harvested in 2019).

## Data Availability

All data generated or analyzed during this study are included in this published article and its supplementary information files.

## Abbreviations

CBB: Coomassie brilliant blue
DAF: Days after flowering
ER: Endoplasmic reticulum
LM: Light microscopy
LC-MS/MS: Liquid chromatography-tandem mass spectrometry
PAS: Periodic acid–Schiff
PBs: Protein bodies
PB-I: Protein body I
PB-II: Protein body II
PCR: Polymerase chain reaction
PDIL: Protein disulfide isomerase-like protein
PSV: Protein storage vacuole
qRT-PCR: Quantitative real-time PCR
SDS-PAGE: Sodium dodecyl sulfate-polyacrylamide gel electrophoresis
SEM: Scanning electron microscopy
SSPs: Seed storage proteins
TEM: Transmission electron microscopy
TMT: Tandem mass tags
WGD: Whole-genome duplication
