# Supplementary material for: Unique Glutelin Expression Patterns and Seed Endosperm Structure Facilitate Glutelin Accumulation in Polyploid Rice Seed
Source: Rice (N Y). 2021 Jul 5;14:61. doi: 10.1186/s12284-021-00500-0 (PMC8257881; doi:10.1186/s12284-021-00500-0)
Supplement: Supplementary file 1 — Additional file 1: Figure S1. Phylogenic relationships among rice glutelin proteins. A rooted tree was generated based on a multiple sequence alignment using MEGA X. Glutelins were grouped into four distinct sub-clades (GluA, GluB, GluC and GluD). Figure S2. Expression analysis of SSPs using gradient SDS-PAGE (4%–20%). CBB staining of total protein in five pairs of mature rice seeds (NJ11-2x and NJ11-4x, CX35-2x and CX35-4x, Mudgo-2x and Mudgo-4x, HJK-2x and HJK-4x, and Balilla-2x and Balilla-4x, harvested in 2019). Each lane contains rice grains of same weight. M = molecular size marker. Proteins were extracted from rice seeds at 25 DAF. Pro-glutelin polypeptides, glutelin acidic and basic subunits, and prolamins are indicated by black vertical lines. Figure S3. Variations in aleurone layer structural characterization in rice seeds with various ploidy levels. SEM images of aleurone layers and endosperms of 9311-2x and 9311-4x seeds at 17 and 25 DAF (harvested in 2018). Scale bar: 50 μm. Figure S4. Variations in starchy endosperm structural characterization in rice seeds with various ploidy levels. SEM images of seed endosperms in two pairs of rice lines (9311-2x and 9311-4x, and A3-2x and A3-4x, harvested in 2018) at 25 DAF. Scale bar: 10 μm, 50 μm and 500 μm. [file 12284_2021_500_MOESM1_ESM.doc]

**
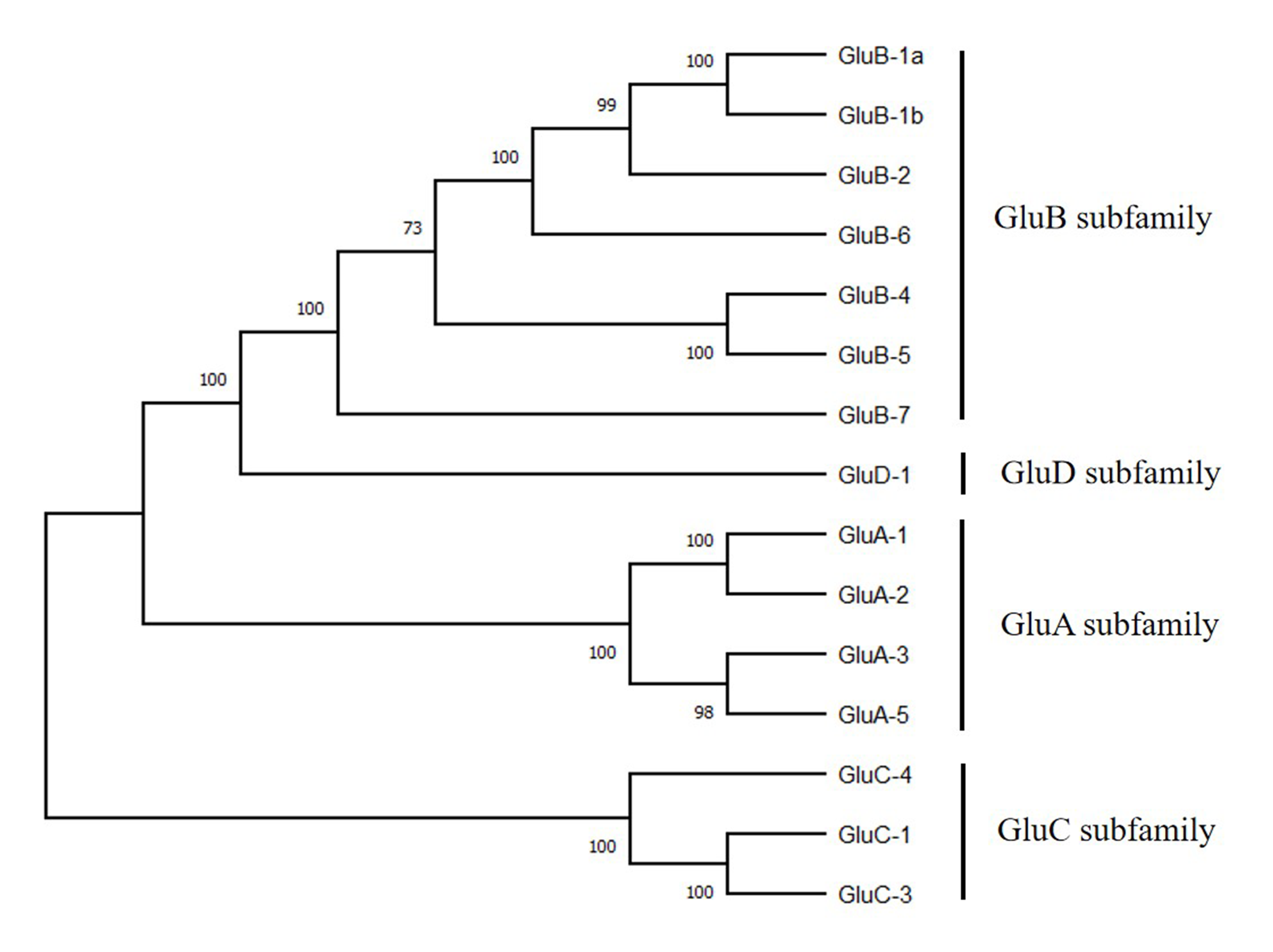
**

**Figure S1.** Phylogenic relationship among rice glutelin proteins. A rooted tree was generated based on a multiple sequence alignment using MEGA X. Glutelins were grouped into four distinct sub-clades (*GluA, GluB, GluC and GluD*).


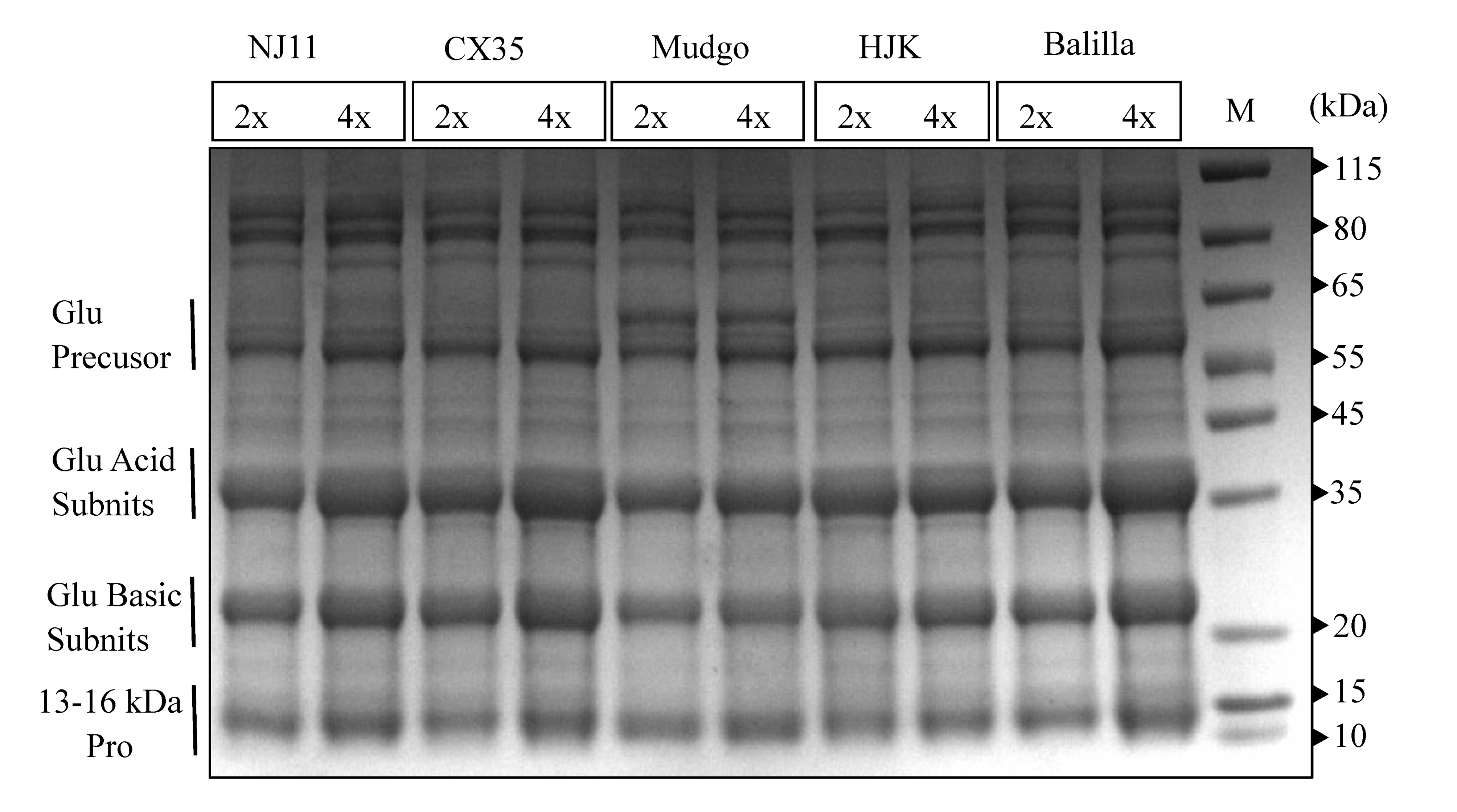


**Figure S2**. Expression analysis of SSPs using gradient SDS-PAGE (4–20%). CBB staining of total protein in five pairs of rice mature seeds (NJ11-2x and NJ11-4x, CX35-2x and CX35-4x, Mudgo-2x and Mudgo-4x, HJK-2x and HJK-4x, and Balilla-2x and Balilla-4x, harvested in 2019). Each lane contains rice grains of same weight. M=molecular size marker. Proteins were extracted from rice seeds at 25 DAF. Pro-glutelin polypeptides, glutelin acidic and basic subunits, and prolamins are indicated by black vertical lines.


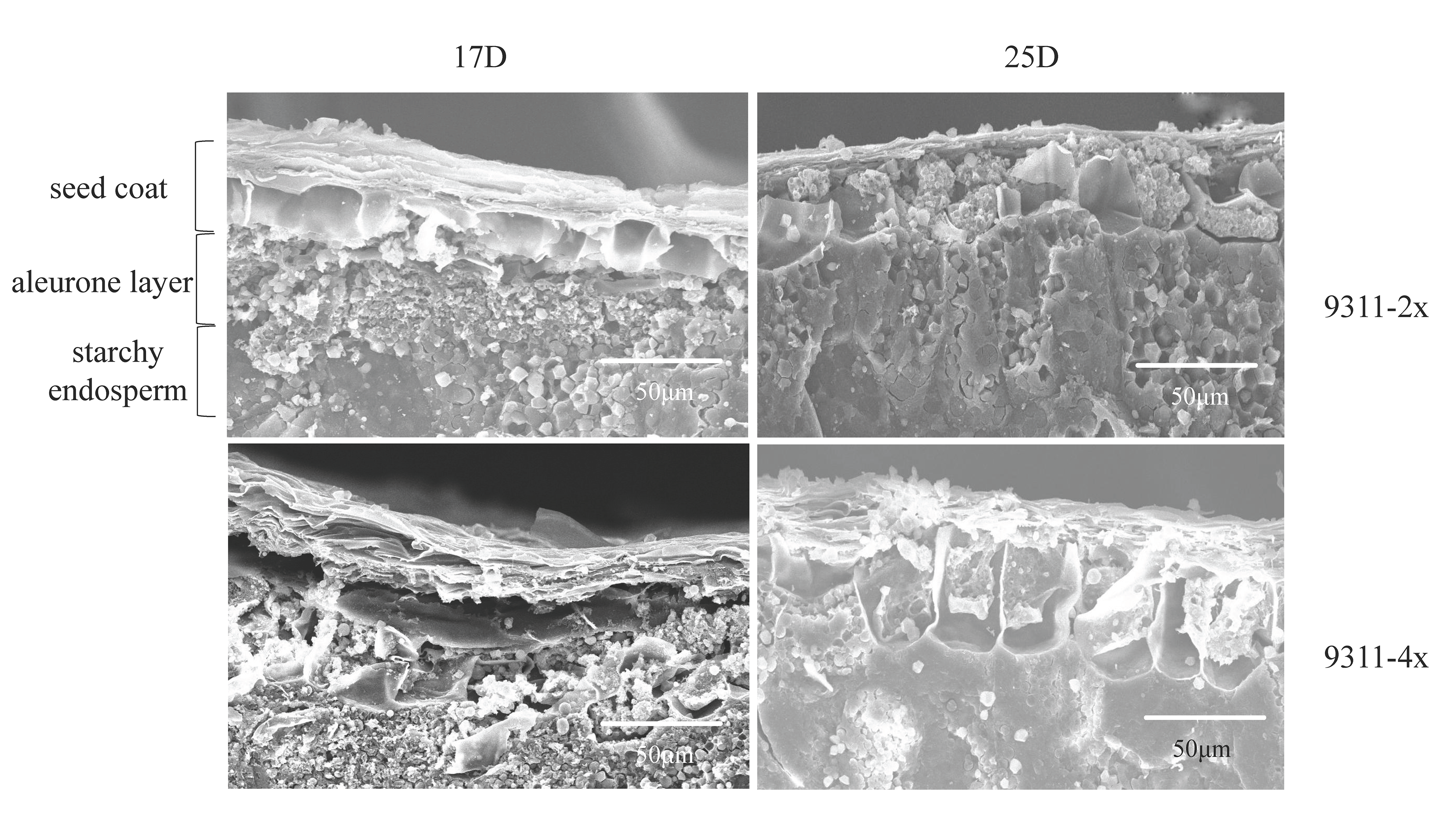


**Figure S3**. Variations in aleurone layer structural characterization in rice seeds with various ploidy levels. SEM images of aleurone layers and endosperms of 9311-2x and 9311-4x seeds at 17 and 25 DAF (harvested in 2018). Scale bar: 50 μm.


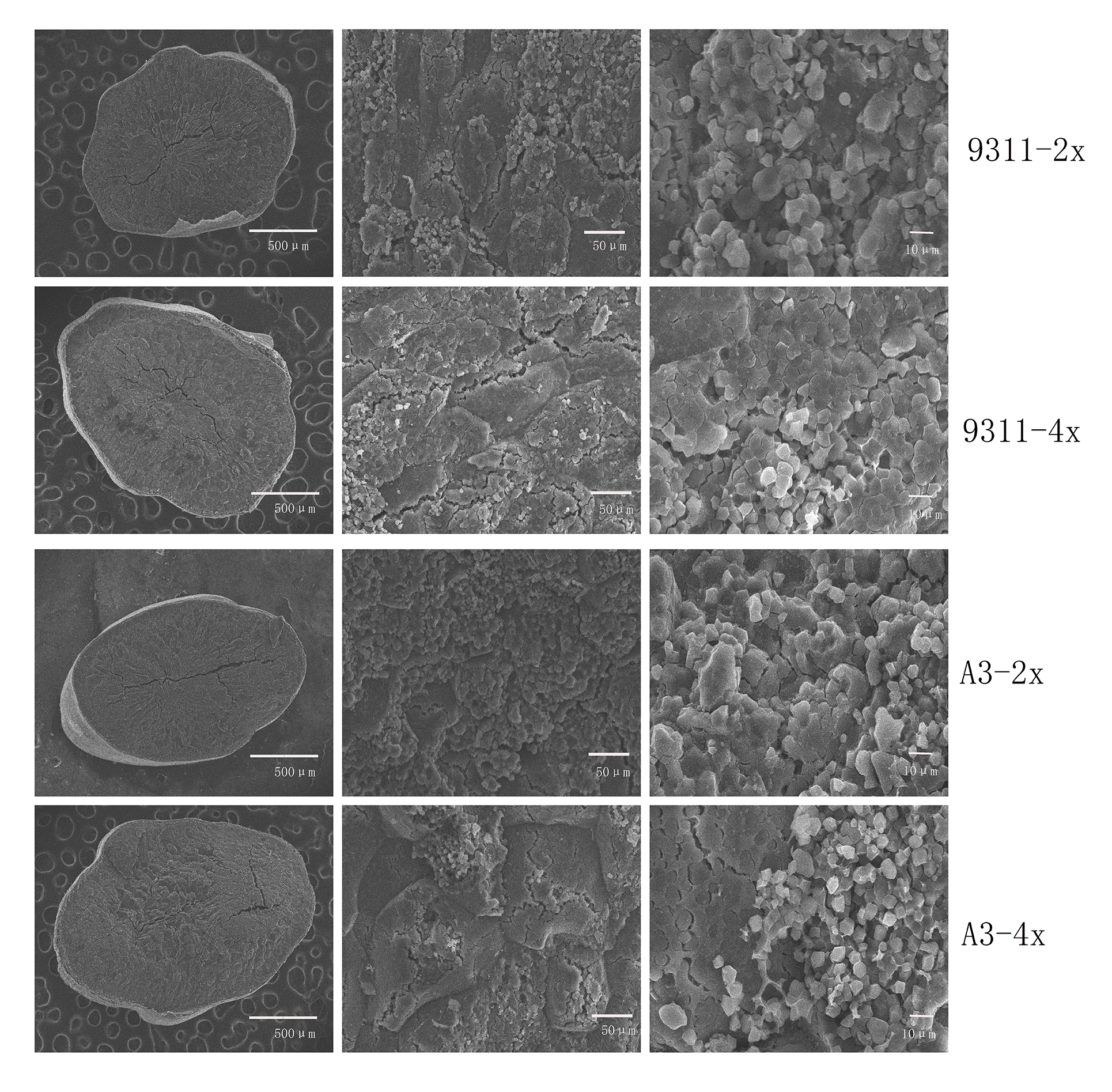


**Figure S4.** Variations in starchy endosperm structural characterization in rice seeds with various ploidy levels. SEM images of seed endosperms in two pairs of rice lines (9311-2x and 9311-4x, and A3-2x and A3-4x, harvested in 2018) at 25 DAF. Scale bar: 10 μm, 50 μm and 500 μm.
