# Supplementary material for: Unique Glutelin Expression Patterns and Seed Endosperm Structure Facilitate Glutelin Accumulation in Polyploid Rice Seed
Source: Rice (N Y). 2021 Jul 5;14:61. doi: 10.1186/s12284-021-00500-0 (PMC8257881; doi:10.1186/s12284-021-00500-0)
Supplement: Supplementary file 2 — Additional file 2: Table S1. Classification of rice glutelin genes. Table S2. Total protein content and average growth of tetraploid brown rice seeds and the corresponding diploid rice seeds harvested in Wuhan between November 2018 and November 2019. Data are presented as means ± standard errors of three biological replicates; * denotes significant differences in protein content between tetraploid and diploid rice (p < 0.05); ** denotes extremely significant differences (p < 0.01). Table S3. Glutelin and prolamin contents in tetraploid and diploid rice seeds (harvested in 2019). * denotes significant differences in component protein content between tetraploid and diploid rice (p < 0.05); ** denotes extremely significant differences (p < 0.01). Table S4. Seventeen amino acid contents of tetraploid and the corresponding diploid brown rice seeds (harvested in 2019). Data are presented means ± standard errors of three biological replicates; * denotes significant differences in amino acid contents between tetraploid and the corresponding diploid rice (p < 0.05); ** denotes extremely significant differences (p < 0.01). Table S5. Amylose contents of tetraploid and diploid rice (harvested in 2019). Data are presented as means ± standard errors of three biological replicates. * denotes significant differences in amylose contents between tetraploid and diploid rice (p < 0.05). ** denotes extremely significant differences (p < 0.01). Table S6. Synthetic peptides for polyclonal anti-glutelin antibodies. Table S7. Primer sequences used for qRT-PCR. [file 12284_2021_500_MOESM2_ESM.doc]

**Table S1.** Classification of rice glutelin genes.

| **Gene name** | **Classifification** | **Locus name** | | **Chromosome** | **cDNA clone^c^** |
| --- | --- | --- | --- | --- | --- |
|  |  | **TIGR-osa1^a^** | **RAP-DB^b^** |  |  |
| *GluA-1* | GluA | LOC_Os01G55690 | Os01G0762500 | 1 | pREE61,pRG206 |
| *GluA-2* | GluA | LOC_Os10G26060 | Os10G0400200 | 10 | pREE77 |
| *GluA-3* | GluA | LOC_Os03G31360 | Os03G0427300 | 3 | pG22,pREE79 |
| *GluA-4* | GluA | PseudoGene | PseudoGene | 1 |  |
| *GluA-5* | GluA | LOC_Os01G55630 | [Os01G0761800](http://rapdb.dna.affrc.go.jp/viewer/gbrowse_details/irgsp1?name=Os01g0761800) | 1 |  |
| *GluB-1a* | GluB | LOC_Os02G15169 | Os02G0249800 | 2 | pREEK1 |
| *GluB-1b* | GluB | LOC_Os02G15178 | Os02G0249900 | 2 |  |
| *GluB-2* | GluB | LOC_Os02G15150 | Os02G0249600 | 2 | GluB-2 |
| *GluB-3* | GluB | PseudoGene | PseudoGene | 2 |  |
| *GluB-4* | GluB | LOC_Os02G16830 | Os02G0268300 | 2 | λRG21,λRG55 |
| *GluB-5* | GluB | LOC_Os02G16820 | Os02G0268100 | 2 |  |
| *GluB-6* | GluB | LOC_Os02G15070 | Os02G0248800 | 2 | GluB-6 |
| *GluB-7* | GluB | LOC_Os02G14600 | Os02G0242600 | 2 | GluB-7 |
| *GluC-1* | GluC | LOC_Os02G25640 | Os02G0453600 | 2 |  |
| *GluC-2* | GluC | PseudoGene | PseudoGene | 2 |  |
| *GluC-3* | GluC | LOC_Os02G25860 | [Os02G0456100](http://rapdb.dna.affrc.go.jp/viewer/gbrowse_details/irgsp1?name=Os02g0456100) | 2 |  |
| *GluC-4* | GluC | LOC_Os08G03410 | Os08G0127900 | 8 |  |
| *GluD-1* | GluD | LOC_Os02G15090 | Os02G0249000 | 2 | GluD-1 |

^a^The Institute for Genome Research_rice genome annotation database(osa1) (http://blast.jcvi.org/euk-blast/index.cgi?project=osa1).

^b^The Rice Annotation Project Database (http://rapdb.dna.affrc.go.jp).

^c^cDNA clones of Oryza sativa were previously identified by research institutions.

Table S2. Total protein content and average growth of tetraploid brown rice seeds and the corresponding diploid rice seeds harvested in Wuhan between November 2018 and November 2019.

| Rice Variety | Crop year 2018 | | | Crop year 2019 | | |
| --- | --- | --- | --- | --- | --- | --- |
|  | Protein content(mg/100mg) | Average increase amplitude(%) | P-value | Protein content (mg/100mg) | Average increase  amplitude(%) | P-value |
| 9311-2x | 9.79±0.82 |  |  | 8.37±0.80 |  |  |
| 9311-4x | 13.42±0.97 | 37.21±1.88** | 0.007774 | 13.15±0.93 | 57.33±4.05** | 0.002511 |
| A3-2x | 9.07±0.67 |  |  | 7.76±0.54 |  |  |
| A3-4x | 11.08±0.53 | 22.30±3.33* | 0.015330 | 9.77±0.64 | 25.90±3.32* | 0.014532 |
| Mudgo-2x | 8.14±0.55 |  |  | 7.96±0.67 |  |  |
| Mudgo-4x | 10.84±0.78 | 34.03±1.86** | 0.008001 | 10.29±0.65 | 30.31±1.86* | 0.012429 |
| Balilla-2x | 9.14±0.52 |  |  | 8.28±0.79 |  |  |
| Balilla-4x | 10.79±0.59 | 18.02±0.80* | 0.022419 | 9.95±0.36 | 20.64±8.26* | 0.029691 |
| NJ11-2x | 9.25±0.64 |  |  | 9.44±0.78 |  |  |
| NJ11-4x | 11.24±0.50 | 21.65±3.03* | 0.013383 | 11.85±0.75 | 21.64±3.19* | 0.018163 |
| HJK-2x | 8.90±0.38 |  |  | 8.75±0.32 |  |  |
| HJK-4x | 10.08±0.46 | 13.33±1.44* | 0.026953 | 9.96±0.53 | 13.87±1.90* | 0.027334 |
| CX22-2x | 7.75±0.34 |  |  | 8.20±0.41 |  |  |
| CX22-4x | 9.42±0.63 | 21.48±3.27* | 0.015469 | 9.91±0.60 | 20.91±2.55* | 0.015216 |
| CX27-2x | 9.22±0.51 |  |  | 8.46±0.42 |  |  |
| CX27-4x | 10.54±0.39 | 14.32±2.60* | 0.024770 | 9.89±0.76 | 16.80±3.20* | 0.046873 |
| CX35-2x | 7.47±0.72 |  |  | 8.52±0.43 |  |  |
| CX35-4x | 10.6±0.98 | 41.91±3.56* | 0.011398 | 10.85±0.54 | 27.30±0.15** | 0.004275 |
| CX46-2x | 6.69±0.64 |  |  | 7.31±0.36 |  |  |
| CX46-4x | 9.63±0.70 | 44.03±4.54** | 0.005960 | 9.75±0.29 | 33.46±2.78** | 0.000801 |
| CX101-2x | 9.84±0.55 |  |  | 8.73±0.57 |  |  |
| CX101-4x | 11.48±0.68 | 16.76±3.93* | 0.031916 | 10.64±0.54 | 21.87±2.07* | 0.013829 |
| CX753-2x | 8.56±0.35 |  |  | 7.67±0.28 |  |  |
| CX753-4x | 10.81±0.45 | 26.25±0.91** | 0.002448 | 9.74±0.35 | 26.93±0.51** | 0.001320 |
| CQ-2x | 9.42±0.73 |  |  | 9.18±0.38 |  |  |
| CQ-4x | 10.83±0.48 | 15.26±4.61* | 0.048376 | 10.89±0.79 | 18.55±4.43* | 0.027927 |
| DD25-2x | 8.44±0.36 |  |  | 9.26±0.35 |  |  |
| DD25-4x | 11.75±0.52 | 39.16±0.13** | 0.000826 | 11.71±0.48 | 26.44±0.90** | 0.002069 |
| GY05-2x | 10.68±0.37 |  |  | 9.24±0.43 |  |  |
| GY05-4x | 12.37±0.47 | 15.82±3.53** | 0.008396 | 11.84±0.33 | 28.17±3.15** | 0.001146 |
| GY24-2x | 11.15±0.59 |  |  | 10.52±0.54 |  |  |
| GY24-4x | 13.31±0.75 | 19.36±0.79* | 0.017332 | 12.78±0.65 | 21.56±1.74** | 0.009645 |
| GY36-2x | 9.27±0.40 |  |  | 10.21±0.40 |  |  |
| GY36-4x | 11.62±0.53 | 25.42±3.76** | 0.003517 | 11.95±0.23 | 17.19±6.93** | 0.002819 |
| GY37-2x | 8.44±0.54 |  |  | 9.07±0.48 |  |  |
| GY37-4x | 10.51±0.79 | 22.63±2.06* | 0.019976 | 10.94±0.78 | 20.58±2.33* | 0.023886 |
| GY53-2x | 9.10±0.76 |  |  | 9.64±0.49 |  |  |
| GY53-4x | 13.13±0.63 | 44.67±5.48** | 0.002108 | 12.65±0.53 | 31.27±2.42** | 0.001992 |
| KX170-2x | 9.25±0.69 |  |  | 8.45±0.56 |  |  |
| KX170-4x | 11.86±0.36 | 28.53±6.73** | 0.004420 | 10.40±0.75 | 23.09±2.09* | 0.022490 |
| KX171-2x | 10.59±0.53 |  |  | 9.38±0.62 |  |  |
| KX171-4x | 11.86±0.49 | 12.05±3.07* | 0.037750 | 12.08±0.88 | 28.79±2.02* | 0.012320 |
| T1-2x | 8.58±0.70 |  |  | 9.43±0.35 |  |  |
| T1-4x | 9.58±0.54 | 11.81±2.77 | 0.122109 | 10.73±0.72 | 13.78±3.31* | 0.047739 |
| TGXM-2x | 9.40±0.85 |  |  | 8.76±0.67 |  |  |
| TGXM-4x | 11.26±0.44 | 20.34±7.76* | 0.027648 | 10.64±0.87 | 21.52±1.65* | 0.041237 |
| YZ32-2x | 8.32±0.35 |  |  | 9.21±0.43 |  |  |
| YZ32-4x | 9.63±0.58 | 15.70±5.20* | 0.029273 | 10.32±0.60 | 11.97±1.23 | 0.060414 |

Data are means ± standard errors of three biological replicates;*: Shows significant differences of protein content between the tetraploid rice and its diploid rice (p < 0.05); **: Shows extremely significant differences (p < 0.01).

**Table S3.** Glutelin and prolamin contents in tetraploid and diploid rice seeds (harvested in 2019).

| **Rice Variety** | **Glutelin content**  **（mg/100mg）** | **Average increase amplitude(%)** | **P-value** |
| --- | --- | --- | --- |
| 9311-2x | 2.18±0.04 |  |  |
| 9311-4x | 3.54±0.139 | 62.73±4.46** | 0.000050 |
| A3-2x | 1.94±0.18 |  |  |
| A3-4x | 2.64±0.12 | 35.99±6.68** | 0.005400 |
| Mudgo-2x | 2.15±0.04 |  |  |
| Mudgo-4x | 2.77±0.12 | 29.27±8.16** | 0.001091 |
| Balilla-2x | 2.04±0.12 |  |  |
| Balilla-4x | 2.81±0.12 | 37.22±4.49** | 0.001411 |
| NJ11-2x | 2.37±0.07 |  |  |
| NJ11-4x | 2.61±0.04 | 10.32±2.54** | 0.006058 |
| HJK-2x | 1.85±0.13 |  |  |
| HJK-4x | 2.21±0.03 | 19.75±7.19* | 0.010900 |

| **Rice Variety** | **Prolamin content**  **（mg/100mg）** | **Average increase amplitude(%)** | **P-value** |
| --- | --- | --- | --- |
| 9311-2x | 0.44±0.02 |  |  |
| 9311-4x | 0.49±0.02 | 10.73±2.29* | 0.033471 |
| A3-2x | 0.46±0.03 |  |  |
| A3-4x | 0.52±0.03 | 13.15±3.29* | 0.040706 |
| Mudgo-2x | 0.47±0.06 |  |  |
| Mudgo-4x | 0.48±0.04 | 2.77±6.00 | 0.402179 |
| Balilla-2x | 0.40±0.02 |  |  |
| Balilla-4x | 0.45±0.02 | 11.72±1.92* | 0.032615 |
| NJ11-2x | 0.37±0.06 |  |  |
| NJ11-4 | 0.42±0.04 | 15.31±8.23 | 0.242249 |
| HJK-2x | 0.49±0.02 |  |  |
| HJK-4x | 0.59±0.03 | 18.71±2.25* | 0.014896 |

*: Denotes significant differences in component protein content between tetraploid and diploid rices (p < 0.05); **: Denotes extremely significant differences (p < 0.01).

**Table S4.** Seventeen amino acids contents of tetraploid and the corresponding diploid brown rice seeds (harvested in 2019).

| **Amino Acid** | **9311-2x** | **9311-4x** | **Average increase amplitude(%)** | **P-value** | **A3-2x** | **A3-4x** | **Average increase amplitude(%)** | **P-value** |
| --- | --- | --- | --- | --- | --- | --- | --- | --- |
| Lys | 0.31±0.04 | 0.46±0.04 | 49.90±5.72** | 0.007130 | 0.30±0.03 | 0.39±0.02 | 31.96±6.80* | 0.011778 |
| Thr | 0.28±0.02 | 0.44±0.02 | 56.94±4.95** | 0.000395 | 0.26±0.04 | 0.35±0.04 | 35.46±5.03* | 0.042562 |
| Met | 0.11±0.03 | 0.19±0.04 | 75.89±10.79* | 0.030627 | 0.06±0.02 | 0.11±0.03 | 89.12±9.63* | 0.043638 |
| Ile | 0.31±0.03 | 0.50±0.04 | 63.80±3.97** | 0.001436 | 0.29±0.03 | 0.41±0.03 | 39.74±4.08** | 0.005691 |
| Val | 0.49±0.04 | 0.75±0.03 | 54.63±4.99** | 0.000697 | 0.44±0.03 | 0.51±0.03 | 39.78±6.79** | 0.001983 |
| Leu | 0.72±0.03 | 1.17±0.05 | 63.96±7.54** | 0.000139 | 0.64±0.02 | 0.91±0.03 | 42.72±6.31** | 0.000190 |
| Phe | 0.50±0.04 | 0.75±0.04 | 51.56±2.96** | 0.001451 | 0.49±0.03 | 0.59±0.03 | 22.04±2.48* | 0.010027 |
| His | 0.21±0.04 | 0.32±0.04 | 53.94±9.24* | 0.023937 | 0.17±0.02 | 0.25±0.03 | 43.31±6.42* | 0.024411 |
| Arg | 0.60±0.02 | 0.97±0.05 | 62.40±6.81** | 0.000187 | 0.56±0.02 | 0.76±0.03 | 36.84±9.80** | 0.000714 |
| Cys | 0.20±0.04 | 0.34±0.04 | 71.90±11.96* | 0.010285 | 0.15±0.02 | 0.19±0.02 | 71.90±11.96 | 0.058039 |
| Asp | 0.75±0.02 | 1.19±0.03 | 58.54±5.28** | 0.000022 | 0.71±0.04 | 0.99±0.06 | 39.97±9.72** | 0.002139 |
| Ser | 0.47±0.05 | 0.72±0.05 | 51.47±6.88** | 0.004797 | 0.40±0.03 | 0.55±0.05 | 51.47±6.88** | 0.008442 |
| Glu | 1.53±0.07 | 2.52±0.03 | 64.50±6.44** | 0.000019 | 1.37±0.04 | 1.97±0.05 | 44.29±4.23** | 0.000084 |
| Gly | 0.35±0.02 | 0.57±0.01 | 61.08±8.13** | 0.000170 | 0.34±0.03 | 0.48±0.03 | 41.29±6.75** | 0.005253 |
| Ala | 0.50±0.02 | 0.74±0.04 | 50.05±4.24** | 0.000722 | 0.46±0.03 | 0.60±0.03 | 31.56±3.18** | 0.002577 |
| Tyr | 0.25±0.02 | 0.38±0.04 | 51.85±5.26** | 0.004207 | 0.36±0.03 | 0.29±0.02 | -19.80±7.35* | 0.026478 |
| Pro | 0.26±0.03 | 0.41±0.04 | 57.87±5.79** | 0.006010 | 0.27±0.02 | 0.35±0.03 | 27.23±7.44* | 0.020488 |
| Total（%） | 7.84±0.21 | 12.45±0.22 | 58.80±1.86** | 0.000012 | 7.25±0.18 | 9.80±0.32 | 35.20±4.41** | 0.000277 |

Data are means ± standard errors of three biological replicates;*: Shows significant differences of amino acid contents between the tetraploid and its corresponding diploid rice(p < 0.05); **: Shows extremely significant differences (p < 0.01)

**Table S5.** Amylose contents of tetraploid and diploid rice (harvested in 2019).

| Rice Variety | Amyloses content (mg/100mg) | Average decrease amplitude(%) | P-value |
| --- | --- | --- | --- |
| 9311-2x | 15.41±0.50 |  |  |
| 9311-4x | 13.57±0.48 | 11.8±5.81** | 0.009872 |
| A3-2x | 15.11±0.34 |  |  |
| A3-4x | 14.86±0.37 | 16.15±3.58 | 0.438350 |
| Mudgo-2x | 16.18±0.28 |  |  |
| Mudgo-4x | 14.63±0.36 | 9.56±2.78** | 0.004206 |
| Balilla-2x | 13.76±0.42 |  |  |
| Balilla-4x | 11.73±0.39 | 14.77±0.47** | 0.003429 |
| NJ11-2x | 15.28±0.45 |  |  |
| NJ11-4x | 13.32±0.37 | 12.82±0.45** | 0.004420 |
| HJK-2x | 15.01±0.59 |  |  |
| HJK-4x | 14.64±0.75 | 2.47±1.39 | 0.542447 |

Data are presented as means ± standard errors of three biological replicates.*: denotes significant differences in amylose contents between tetraploid and diploid rice (p < 0.05).**: denotes extremely significant differences (p < 0.01).

**Table S6. Synthetic peptides for polyclonal anti-glutelin antibodies.**

| **Gene** | **Peptide sequence** | **Accession No.** | **Positions** | **Reference** |
| --- | --- | --- | --- | --- |
| Anti-GLUA-1 | EQGQVQS | X05661 | 280-286 | He et al., 2013 |
| Anti-GLUA-2 | KRNPQAYRREVEEWSQ | X05664 | 210-225 | Takahashi etal.,2019 |
| Anti-GLUB-1 | QVQYSERQQTSSRW | X54314 | 288-301 | Takahashi etal.,2019 |
| Anti-GLUB-2 | RVQQVYGSS | X54192 | 207-215 | He et al., 2013 |
| Anti-GLUB-4/5 | KLLRPAFA | CAA32566 | 265-272 | He et al., 2013 |
| Anti-GLUC-1 | LQSPRGFRGDQDS | AB016501 | 29-44 | Takahashi etal.,2019 |
| Anti-GLUD-1 | TQRQEEHRQYQQVQYR | AY429650 | 265-280 | Kawakatsu et al., 2008; Takahashi etal.,2019 |

Table S7. Primer sequences used for qRT-PCR.

| **Gene** | **Forward primer** | **Reverse primer** | **Accession No.** |
| --- | --- | --- | --- |
| *GluA-1* | AAGCATACAGGCGTGAGGTT | GGTCATTTTGACATTGGAGC | Os01g0762500 |
| *GluA-2* | GAGCATTATCAAGAAGGAGG | TCAGCACGGTTAGGATTAT | Os10g0400200 |
| *GluB-1* | ATTCCGTGCCTTGCCAG | CTCTGAGGTCTCGCTTTCG | Os02g0249800 |
| *GluB-2* | TGAAGAAAGCAGAGCGTG | CGTGAGATGCGGTAAGC | Os02g0249600 |
| *GluC-1* | CACAAGGGCCAATAGCCAGA | GGTCACGTACATCACCGTGT | Os02g0453600 |
| *GluD-1* | AAGACAGAGCGACCAAGCTC | ATGTGCAACACTAGCCGGAA | Os02g0249000 |
| *PDI1-1* | CCGATTGTTCTTGCCAAGGTTG | TCCTGAATGTTCTTGCCCTG | Os11g0199200 |
| *β-ACTIN* | TGACGGAGCGTGGTTAC | GAGGAGCTGGTCTTGGC | Os03g0718100 |
| *Ubiquitin* | TCCGTGGTGGTCAGTAATCA | ACTGCTGTCCCACAGGAAAC | Os02g0161900 |
